# Prostate Cancer Incidence, Mortality, and Survival in Switzerland

**DOI:** 10.1001/jamanetworkopen.2026.8289

**Published:** 2026-04-21

**Authors:** Dominik Menges, Lea Wildisen, Thomas Scherer, Stefano Tancredi, Christoph Würnschimmel, Silvan Sigg, Shuang Hao, Max Lippuner, Richard Cathomas, Arnoud J. Templeton, Corinne Chmiel, Cyrill A. Rentsch, Daniel Eberli, Maciej Kwiatkowski, Ashkan Mortezavi, Arnaud Chiolero, Sabine Rohrmann, Mark Clements, Katharina Staehelin

**Affiliations:** 1 Epidemiology, Biostatistics and Prevention Institute, University of Zurich, Zurich, Switzerland; 2 Department of Medical Epidemiology and Biostatistics, Karolinska Institutet, Stockholm, Sweden; 3 Swiss School of Public Health, Zurich, Switzerland; 4 National Agency for Cancer Registration, Zurich, Switzerland; 5 National Institute for Cancer Epidemiology and Registration, Zurich, Switzerland; 6 Department of Urology, University Hospital Zurich, University of Zurich, Zurich, Switzerland; 7 Population Health Laboratory, University of Fribourg, Fribourg, Switzerland; 8 University Teaching and Research Hospital, Luzerner Kantonsspital, Lucerne, Switzerland; 9 Faculty of Health Sciences and Medicine, University of Lucerne, Lucerne, Switzerland; 10 Europa Uomo Switzerland, Ehrendingen, Switzerland; 11 Division of Medical Oncology and Hematology, Cantonal Hospital Graubünden, Chur, Switzerland; 12 Department of Medical Oncology, St Claraspital, Basel, Switzerland; 13 St Clara Research Ltd, Basel, Switzerland; 14 Faculty of Medicine, University of Basel, Basel, Switzerland; 15 mediX Schweiz, Zurich, Switzerland; 16 Department of Urology, University Hospital Basel, University of Basel, Basel, Switzerland; 17 Department of Urology, Cantonal Hospital Aarau, Aarau, Switzerland; 18 Department of Urology, Academic Hospital Braunschweig, Braunschweig, Germany; 19 School of Population and Global Health, McGill University, Montreal, Quebec, Canada; 20 Cancer Registry Zurich, Zug, Schaffhausen and Schwyz, University Hospital Zurich, University of Zurich, Zurich, Switzerland

## Abstract

**Importance:**

Prostate cancer (PCa) incidence and prognosis have changed substantially worldwide in recent decades, driven in part by early detection recommendations. Some countries such as the US have shown a rise in metastatic PCa incidence following recommendations against routine PCa screening around 2012.

**Objective:**

To evaluate trends in PCa incidence, mortality, and relative survival across age and prognostic groups and guide policy and practice in Switzerland.

**Design, Setting, and Participants:**

This cohort study included all registered cases of primary PCa based on data from the Swiss National Agency for Cancer Registration and the Swiss Federal Statistical Office from January 1, 1980, to December 31, 2021. Data were analyzed from January 8, 2024, until February 20, 2026.

**Exposure:**

Diagnosis of PCa.

**Main Outcomes and Measures:**

Age-standardized incidence and mortality rates and relative survival to 10 years after diagnosis were estimated. Subgroup analyses were conducted by age, Surveillance, Epidemiology, and End Results stage, Union for International Cancer Control stage, and Gleason score.

**Results:**

The study included 142 665 cases of primary PCa registered in Switzerland between 1980 and 2021, 100 102 (70.2%) of which were men aged 60 to 79 years. From 1980 to 2004, PCa incidence increased to 226.6 (95% CI, 218.8-234.7) per 100 000 men; it decreased markedly to 173.5 (95% CI, 168.9-178.2) per 100 000 men between 2011 and 2014, then increased again to 220.6 (95% CI, 216.0-225.3) per 100 000 men in 2021. These trends were primarily observed for localized, stage I to II PCa and PCa with a Gleason score of 7 or less in men aged 50 to 79 years. Incidence of distant and stage IV PCa increased after 2011, while incidence of PCa with a Gleason score of 8 to 10 remained relatively stable. PCa mortality declined continuously from the early 1990s to 34.8 (95% CI, 32.9-36.8) per 100 000 men in 2021, while 10-year relative survival increased from 46.2% (95% CI, 43.7%-48.8%) in 1982 to 1991 to 88.5% (95% CI, 86.6%-90.5%) in 2012 to 2021. The 10-year relative survival among men with localized PCa (99.2%; 95% CI, 96.0%-102.4%) was similar compared with men from the general population.

**Conclusions and Relevance:**

In this population-based cohort study, trends in PCa incidence in Switzerland suggest that overdiagnosis of lower-risk PCa increased and that there was a concurrent rise in cancers diagnosed in advanced stages alongside improvements in overall prognosis. These findings further suggest substantial potential for optimizing early detection of PCa.

## Key Points

**Question:**

What were the trends in prostate cancer (PCa) incidence, mortality, and relative survival across age and prognostic groups from 1980 to 2021 in Switzerland?

**Findings:**

In this cohort study of 142 665 cases with PCa, incidence increased markedly from 2015 to 2021, particularly lower-risk PCa among men aged 50 to 79 years. Incidence of metastatic PCa increased from 2011 to 2021 following the release of recommendations against routine screening, while prognosis improved and mortality declined from 1980 to 2021.

**Meaning:**

These findings suggest that overdiagnosis of low-risk PCa increased in Switzerland and that there is substantial potential for optimizing early detection of PCa.

## Teaser

This cohort study assesses the incidence, mortality, and relative survival of prostate cancer by age, stage, and Gleason score among men in Switzerland from 1980 to 2021.

## Introduction

The epidemiology of prostate cancer (PCa) is strongly driven by early detection practices and advances in diagnosis and treatment.^1,2^ With PCa being the most frequent cancer in men in most high-income countries,^3^ its incidence globally is projected to increase during the coming decade.^4^ Simultaneously, many diagnosed cancers constitute overdiagnosis and would never become clinically relevant during the men’s lifetime, which current efforts in risk-adapted early detection aim to reduce.^4^ In light of ongoing discussions on organized PCa screening in the European Union and Switzerland,^5,6^ assessing recent trends in PCa incidence and prognosis is crucial to inform public health policy and clinical practice. In-depth country-level evidence can help interpret how early detection measures translate into population-level outcomes and inform decision-making across health systems.

Existing evidence suggests an important influence of prostate-specific antigen (PSA) testing recommendations on PCa incidence and stage at diagnosis. Coinciding with widespread uptake of PSA testing,^1,2^ PCa incidence rapidly increased from the late 1980s until around 2005 to 2010 in many high-income countries.^7,8^ In 2012, the United States Preventive Services Task Force (USPSTF) issued its recommendation against PSA testing for PCa screening due to concerns about overdiagnosis.^9^ This led to relevant decreases in PSA testing^10,11^ and PCa incidence internationally.^7,8^ Corresponding trends in PCa incidence and PSA testing were observed in Switzerland,^12,13^ where the Swiss Medical Board and the Smarter Medicine initiative issued similar recommendations in 2011 and 2014, cautioning against PSA testing without prior shared decision-making.^14,15^ Critically, several studies also reported increases in the incidence of metastatic and high-risk PCa following the 2012 USPSTF recommendations in the US^11,16,17,18,19^ and other countries.^20,21,22^ This raised concerns whether these recommendations may have had adverse consequences.^17^ Despite similarities, PCa incidence trends across high-income countries differ substantially, and population-level risk factors and PSA testing practices may affect stage at diagnosis.^7,8^

In Switzerland, evidence on recent trends in PCa incidence across prognostic groups is currently lacking. Evaluating these trends in the Swiss health care setting with opportunistic PSA testing and recommended shared decision-making may support the interpretation of international trends and provide further evidence on the potential impact of PSA testing recommendations on stage shifts and prognosis. Therefore, this study aimed to assess PCa incidence, mortality, and relative survival by age, Surveillance, Epidemiology, and End Results (SEER) stage, Union for International Cancer Control (UICC) stage, and Gleason score (GS) from 1980 to 2021 in Switzerland.

## Methods

This cohort study did not require ethical approval or informed consent under the Swiss Human Research Act. A declaration of nonresponsibility was obtained from the responsible ethics committee of the canton of Zurich, Switzerland. The study adhered to the Strengthening the Reporting of Observational Studies in Epidemiology (STROBE) reporting guideline.

### Data Sources

This population-based cohort study used Swiss national cancer registration data from January 1, 1980, to December 31, 2021.^23^ Data were obtained from the National Agency for Cancer Registration, which pools data from 13 cancer registries operating at the cantonal level.^24^ Cancer registration in Switzerland has expanded gradually across cantons: while the first registry started in 1969, complete nationwide coverage was only reached from 2020 when the Swiss Cancer Registration Act came into effect (eTable 1 in Supplement 1). We selected 1980 as the starting year to cover approximately 1 decade before introduction of PSA testing. The national mortality dataset from the Swiss Federal Statistical Office (SFSO) from 1980 to 2021 was used for evaluating mortality trends.^25^

We included all primary PCa cases registered between 1980 and 2021. We excluded nonprimary cases (recurrence or progression), cases withdrawing consent for registration, and cases from registries with incomplete case ascertainment in a particular year (1 registry in 2013). For PCa mortality, we used all cases in which PCa (coded according to the *International Classification of Diseases, Eighth Revision*, prior to 1995, and *International Statistical Classification of Diseases, Tenth Revision*, 1995 and later) was registered as the primary cause of death.

### Outcomes and Subgroups

The outcomes of interest were age-standardized PCa incidence rates, age-standardized PCa mortality rates, and 10-year relative survival proportions. We stratified the results by age and prognostic groups, defined as SEER program stage (localized, regional, or distant), UICC stage (I-IV), and GS (≤6, 7, or 8-10). eTable 2 in Supplement 1 provides definitions of stage and GS groups. For prognostic subgroups, we additionally reported proportions across age groups and incidence years. For PCa mortality, age-stratified results were reported.

We conducted sensitivity analyses regarding UICC stage and GS by excluding all cases with prostate surgery as the primary treatment (based on corresponding intervention codes [eTable 3 in Supplement 1]), since nodal status and GS in Swiss cancer registries may be upstaged based on histopathologic assessment of surgical specimen among cases undergoing radical prostatectomy compared with cancers registered based on biopsy alone.

### Statistical Analysis

We performed the statistical analyses following standard methodology used by the National Agency for Cancer Registration and the SFSO.^26^ Analyses were conducted from January 8, 2024, until February 20, 2026. We first estimated the total number of incident PCa cases in Switzerland from those observed across all active registries for years without full registration coverage (1980-2019) by extrapolating within age group, language region, and year, using corresponding population sizes based on SFSO data.^26^ We then used the number of estimated cases for calculating crude and directly age-standardized incidence rates. As registration coverage increased over time, we restricted the analysis to registries active from the beginning of the study time frame in a sensitivity analysis to evaluate the appropriateness of extrapolations in earlier years of registration.

There was relevant missing information regarding stage and GS, which we accounted for by restricting data to 2002 to 2021 and using multiple imputation across primary analyses presented in the article (eMethods in Supplement 1). Further analyses presenting results as registered and based on extrapolation of cases with missing information are reported in the eFigures 1 to 3 in Supplement 1. In prognostic subgroup analyses, we excluded all cases from registries with 100% missingness in specific years or registered during the first year of activity of new registries to reduce the impact of potential data quality issues at initiation of registration. In sensitivity analyses, we applied a more stringent restriction to cases from registries with less than 30% missingness and more than 2 years of registration activity.

We performed direct age standardization for incidence and mortality rates based on the European standard population 2013.^27^ Results based on alternative standard populations are reported in eTables 4 and 5 in Supplement 1. We estimated 95% CIs for age-standardized rates using a normal approximation of log-transformed rates (for results based on multiple imputation) and the method by Fay and Feuer (for all other results).^28,29^ We further calculated changes in age-standardized incidence rates compared with 2011, when Swiss Medical Board recommendations against PCa screening were published.^14^

We estimated relative survival to 10 years after diagnosis using the Ederer II method^30^ and background mortality data from the SFSO.^31^ We excluded all cases registered as death certificate only, with death as reason of detection, or with a follow-up duration of 0 or fewer days (6.2% of all cases) for survival estimation. Since the dataset contained 5-year age strata, we used respective midpoint ages for all strata (ie, 47.5 for <50 years, 52.5 for 50-54 years, 57.5 for 55-59 years, and 87.5 for ≥85 years). Observations were censored at the last known date alive or at 10 years of follow-up. We performed external age-standardization for relative survival estimates based on the International Cancer Survival Standard 1 population.^32,33^ All analyses were conducted in R, version 4.5.2 (R Project for Statistical Computing), using the mice, Epi, and popEpi packages.^34^

## Results

We included 142 665 PCa cases registered between 1980 and 2021 in Switzerland (Table and eFigure 4 in Supplement 1). Most cancers were diagnosed in men aged 60 to 79 years (100 102 [70.2%]).

**Table.** Characteristics of All Included Primary PCa Cases Registered Between 1980 and 2021 in Switzerland

Characteristic	PCa cases, No. (%) (N = 142 665)
Age group, y	
<50	1079 (0.8)
50-59	15 152 (10.6)
60-69	48 150 (33.8)
70-79	51 952 (36.4)
≥80	26 332 (18.5)
Language region^a^	
German speaking	92 645 (64.9)
French or Italian speaking	50 020 (35.1)
Incidence period	
1980-1981	1921 (1.3)
1982-1991	13 465 (9.4)
1992-2001	24 276 (17.0)
2002-2011	37 934 (26.6)
2012-2021	65 069 (45.6)
SEER stage	
Localized	55 814 (62.6)
Regional	22 018 (24.7)
Distant	11 334 (12.7)
Missing^b^	53 499 (37.5)
UICC stage	
I	31 693 (36.0)
II	24 121 (27.4)
III	15 446 (17.5)
IV	16 819 (19.1)
Missing^b^	54 586 (38.3)
Gleason score	
≤6	29 219 (34.1)
7	37 366 (43.6)
8-10	19 117 (22.3)
Missing^b^	56 963 (39.9)

Abbreviations: PCa, prostate cancer; SEER, Surveillance, Epidemiology, and End Results; UICC, Union for International Cancer Control.

^a^ Distribution of cases across language regions corresponded approximately to the respective population sizes (population covered by cancer registration from 1980 to 2021: 62.7% in German-speaking, 37.3% in French- or Italian-speaking regions).

^b^ Proportion of cases with missing information was calculated for the total study period (1980-2021); missingness decreased over time with increasing completeness of cancer registration in Switzerland.

### Incidence

**Figure 1.**
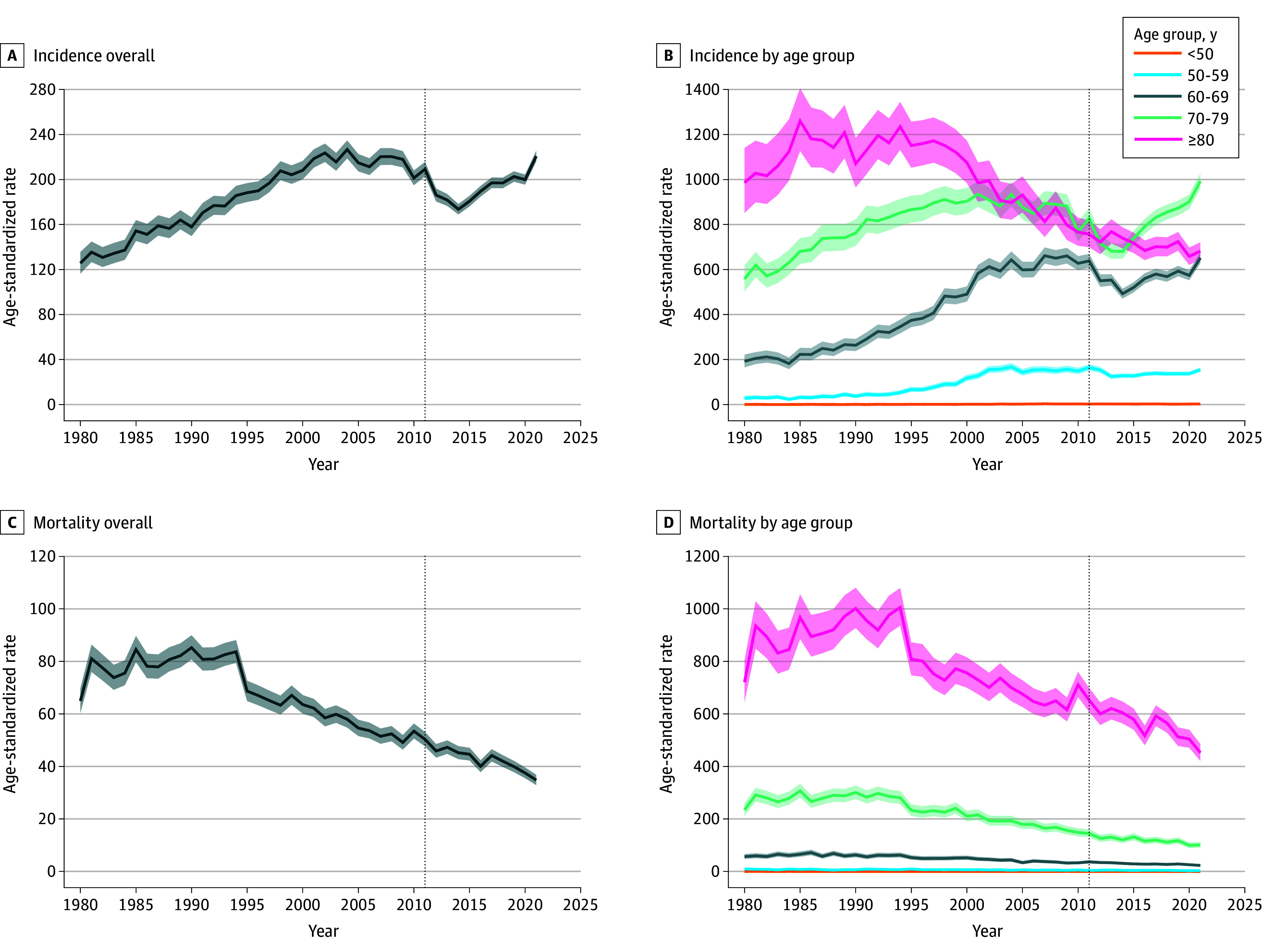
Line Graphs Showing Prostate Cancer Incidence and Mortality Trends Overall and by Age Group Data were accrued from 1980 to 2021. Rates per 100 000 men were directly age standardized using the European 2013 standard population. The vertical dotted line indicates the year 2011, when Swiss Medical Board recommendations against prostate cancer screening were published; shaded areas represent 95% CIs.

Age-standardized PCa incidence rates increased continuously from 125.6 (95% CI, 116.4-135.6) per 100 000 men in 1980 until reaching a peak of 226.6 (95% CI, 218.8-234.7) per 100 000 men in 2004 (Figure 1 and eTables 4 and 6 in Supplement 1). PCa incidence subsequently decreased to its lowest point at 173.5 (95% CI, 168.9-178.2) per 100 000 men in 2014, followed by an increase to 202.6 (95% CI, 198.0-207.2) per 100 000 men in 2019 and a marked increase to 220.6 (95% CI, 216.0-225.3) per 100 000 men in 2021.

Across age groups, PCa incidence among men aged 50 to 79 years generally followed a similar trend as the overall incidence rates, with a marked increase from 2015 to 2021 (Figure 1 and eTables 4 and 6 in Supplement 1). Among men aged 70 to 79 years, PCa incidence reached its highest point in 2021. Meanwhile, it continuously declined between 1997 and 2021 among men 80 years or older, and it was low throughout the study period among men younger than 50 years. Observed incidence trends were similar when restricting to registries active from the beginning of the study time frame (eFigure 5 in Supplement 1).

**Figure 2.**
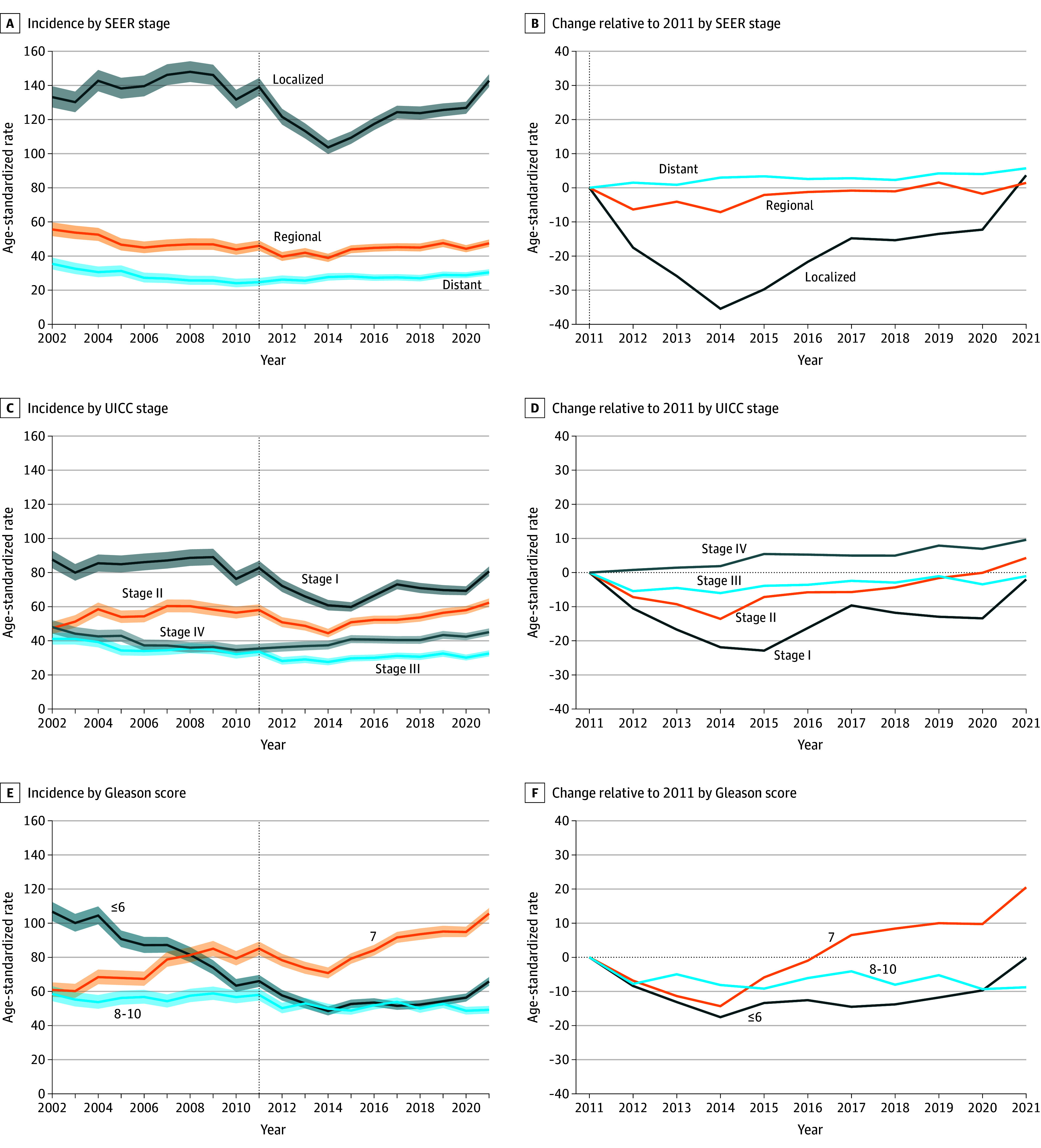
Line Graphs Showing Prostate Cancer Incidence Trends by Surveillance, Epidemiology, and End Results (SEER) Stage, Union for International Cancer Control (UICC) Stage, and Gleason Score Group Data were accrued from 2002 to 2021. Rates per 100 000 men were directly age standardized using the European 2013 standard population. Missing information was imputed using multiple imputation in cases with incomplete registration data. The vertical dotted line indicates the year 2011, when Swiss Medical Board recommendations against prostate cancer screening were published; shaded areas represent 95% CIs.

By SEER stage, localized PCa followed the overall PCa incidence trajectory and showed the highest incidence, followed by regional and distant PCa (Figure 2). The incidence of distant PCa decreased slightly from 2002 to 2011, then subsequently increased again until 2021, with similar trends observed for regional PCa.

By UICC stage, incidence of stages I and II PCa was highest and broadly followed the overall trend, with a peak or plateau between 2007 and 2011, a subsequent decline, and an increase from 2014 to 2021 (Figure 2). The incidence of stages III and IV PCa decreased from 2002 to 2012 before increasing again until 2021.

For GS groups, the incidence of PCa with GS of 7 increased after 2002, with a small, temporary decline between 2011 and 2014 (Figure 2). The incidence of PCa with GS of 6 or less decreased from 2008 to 2014 and increased again in 2018 to 2021. The incidence of PCa with GS of 8 to 10 decreased after 2007 and remained stable from 2012.

Findings based on extrapolation were generally similar to analyses based on multiple imputation (eFigures 1-3 in Supplement 1). In sensitivity analyses excluding cases with prostate surgery to account for potential upstaging, higher incidence rates were observed for UICC stages I and IV PCa, but with similar trends over time compared with primary analyses (eFigure 6 in Supplement 1). Furthermore, we observed a higher incidence of PCa with GS of 6 or less, while trends across GS followed a similar albeit less pronounced trend after 2011 compared with primary analyses. Sensitivity analyses applying tighter restrictions for registry inclusion yielded similar results as the primary analyses (eFigure 7 in Supplement 1).

### Mortality

Overall, age-standardized PCa mortality rates steadily declined from the early 1990s, reaching their lowest point of 34.8 (95% CI, 32.9-36.8) per 100 000 men in 2021 (Figure 1 and eTables 5 and 7 in Supplement 1). Similarly declining trends were observed for all groups of men who were 50 years or older, while PCa mortality among men younger than 50 years remained negligible.

### Relative Survival

**Figure 3.**
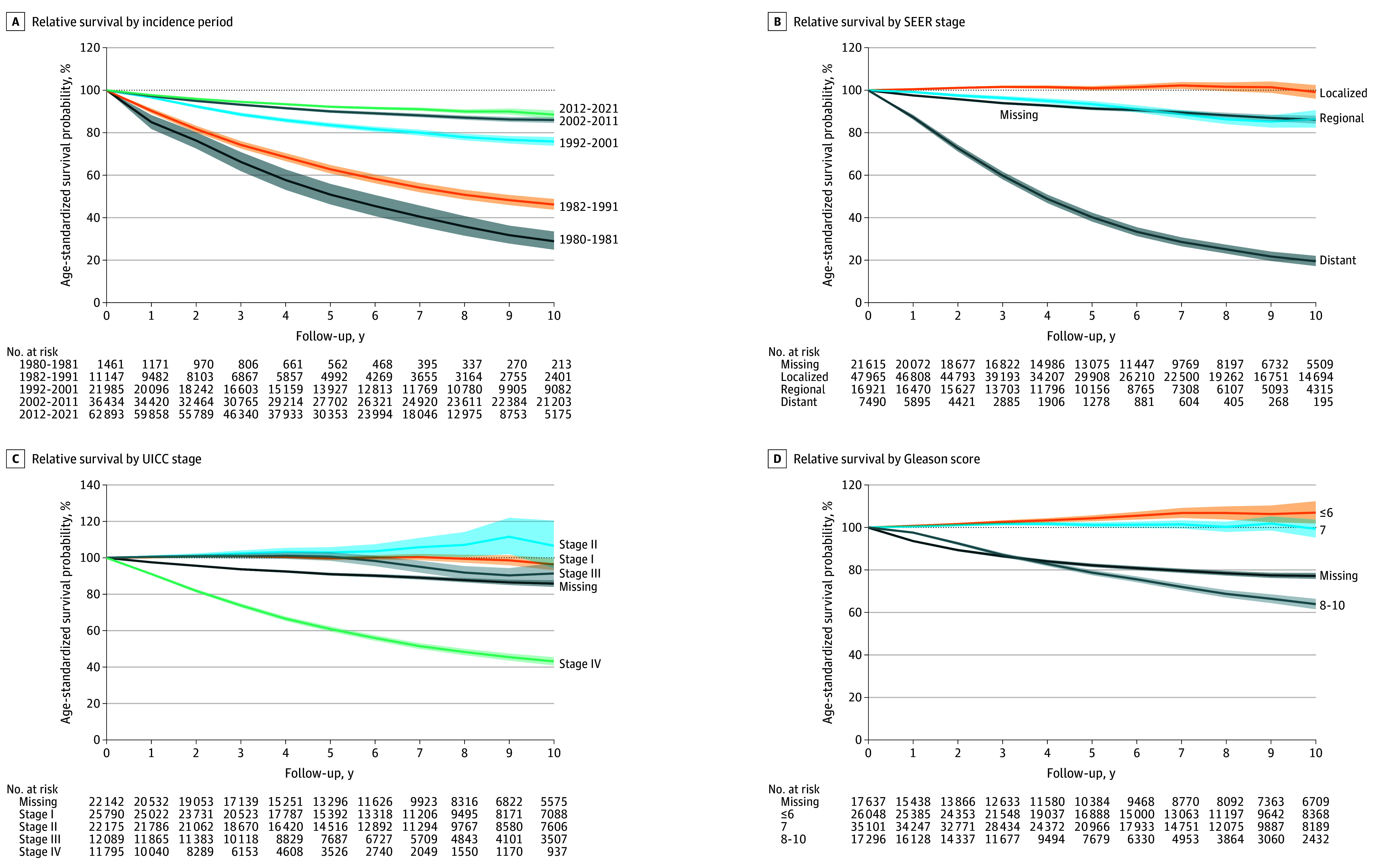
Relative Survival Curves for Prostate Cancer by Incidence Period (1980-2021) and Prognostic Group (2002-2021) Relative survival was estimated relative to the expected mortality among men from the Swiss general population. Relative survival proportions were age-standardized using the International Cancer Survival Standard 1 population. Shaded areas represent 95% CIs. Cases with missing information were treated as a separate category in subgroup analyses. Relative survival proportions greater than 100% represent better survival compared with the general population. SEER indicates Surveillance, Epidemiology, and End Results; UICC, Union for International Cancer Control.

Overall, 10-year relative survival increased over time, from 46.2% (95% CI, 43.7%-48.8%) in 1982 to 1991 to 88.5% (95% CI, 86.6%-90.5%) in 2012 to 2021 (Figure 3 and eTable 8 in Supplement 1). Across age groups, 10-year relative survival was highest among men aged 50 to 59 years (95.6%; 95% CI, 94.5%-96.8%), 60 to 69 years (96.0%; 95% CI, 95.0%-97.0%), and 70 to 79 years (96.2%; 95% CI, 94.4%-98.1%), while it was lower for those younger than 50 years (91.7%; 95% CI, 87.9%-95.7%) and 80 years or older (62.2%; 95% CI, 56.2%-68.9%) in 2012 to 2021.

By SEER stage, men with distant PCa had the poorest prognosis, with a 10-year relative survival of 19.5% (95% CI, 17.2%-22.0%) in 2002 to 2021 (Figure 3 and eFigure 8 in Supplement 1). While 10-year relative survival was 86.4% (95% CI, 82.4%-90.6%) among men with regional PCa, it was 99.2% (95% CI, 96.0%-102.4%) in those with localized PCa. Among men aged 60 to 79 years with localized PCa, relative survival was greater than 100%.

By UICC stage, men with stage I (96.4%; 95% CI, 93.1%-99.7%) and stage II PCa (106.6%; 95% CI, 94.3%-120.4%) had no reduction or only a small reduction in 10-year relative survival (Figure 3 and eFigure 8 in Supplement 1). For stage III, 10-year relative survival was 91.4% (95% CI, 86.0%-97.0%), while it was 43.1% (95% CI, 40.9%-45.4%) for stage IV PCa. Stratified by age, 10-year relative survival was close to or higher than 100% among men aged 60 to 79 years with stage I to III PCa.

Across GS groups, men with PCa with GS of 6 or less and 7 had a 10-year relative survival of 107.0% (95% CI, 101.9%-112.3%) and 99.4% (95% CI, 95.2%-103.7%), respectively, while 10-year relative survival for men with PCa with GS of 8 to 10 was 63.9% (95% CI, 61.5%-66.4%) (Figure 3 and eFigure 8 in Supplement 1). Results were similar across age groups.

Across all subgroup analyses, cases with missing stage or GS information had worse relative survival than the groups with lower-stage or lower-risk PCa (ie, localized, stage I-II, or GS ≤7). Relative survival increased from the 2002-2011 to 2012-2021 periods across all SEER stage groups (eFigure 9 in Supplement 1).

## Discussion

In this population-based study of registered PCa cases from Switzerland, we found an increasing PCa incidence from 1980 until around 2004, a subsequent marked decrease from 2011 to 2014 after recommendations against routine PSA testing were released, and an accelerating increase from 2015 to 2021. These trends were particularly prominent for PCa that was localized, stage I to II PCa, and PCa with GS of 7 or less in men aged 50 to 79 years, with recent increases in PCa incidence primarily observed among men aged 60 to 79 years. Simultaneously, we observed an increase in the incidence of distant and stage IV PCa after 2011; meanwhile, incidence of PCa with GS of 8 to 10 remained relatively stable. From 1980 to 2021, PCa mortality rates declined across all age groups, while 10-year relative survival improved, with men with localized PCa reaching a similar survival as men from the general population.

While the decrease in PCa incidence observed after 2011 in Switzerland is in line with observations from other high-income countries,^7,8^ the marked increase in PCa incidence from 2015 to 2021 stands out and raises concerns regarding potential overdiagnosis. Internationally, the decreasing PCa incidence has primarily been attributed to changes in PSA testing following changes in guidelines, most importantly the USPSTF 2012 recommendations.^10^ A Swiss study has also shown decreased PSA testing following the release of similar Swiss recommendations in 2011 to 2014,^13^ which could explain our observations. Correspondingly, an obvious explanation of the increasing PCa incidence after 2015—especially prominent in men aged 50 to 79 years—is that PSA testing for early detection of PCa may have increased. This hypothesis is also supported by the fact that these trends were primarily observed in PCa that was localized, was stage I or II, and with GS of 7 or less, with increased testing potentially driven by recent developments in using magnetic resonance imaging (MRI) and biomarker-based risk calculators to reduce overdiagnosis.^35,36^ However, external evidence on PSA testing is mixed (eFigure 10 in Supplement 1). In the Swiss Health Survey, there were no substantial increases in the proportion of men aged 55 to 74 years reporting a prostate examination in 2022 relative to 2012 to 2017.^37^ Meanwhile, the proportion of men reporting a prostate examination within the past 12 months in 2022 was higher compared with 2017, indicating more frequent prostate examinations in 2021. The Swiss Healthcare Atlas showed slight increases in PSA testing between 2015 and 2020 (with the caveat that PSA tests may also be prescribed for PCa monitoring), with a marked increase in 2021.^38^ While recent increases in PCa incidence in 2021 could thus be explained through increased testing, explaining prior trends is more challenging.

Further explanations could lie in longer-lasting effects of periods with high rates of PSA testing: reductions in incidence in 2004 to 2014 could be due to the depletion of undiagnosed prevalent PCa cases, with increases after 2015 due to the new formation of tumors in cohorts of men undergoing intensive screening in 2000 to 2010.^39,40^ Additionally, the use of MRI and targeted biopsies (particularly if systematic biopsies are still performed in the absence of relevant MRI findings), lower PSA thresholds, better training of clinicians performing biopsies, or double assessment in pathology may result in greater sensitivity and increased PCa incidence. Overall, we consider it most likely that a combination of these factors is responsible for the observed trends. With discussions ongoing whether PCa with a GS of 6 should be considered cancer in the first place,^41^ it is likely that overdiagnosis is present in Switzerland, given that rates of PCa with GS of 7 increased, PCa with GS of 6 or less returned to 2011 levels after declining until 2014, and PCa with GS of 8 to 10 PCa remained stable from 2012 to 2021.

The increase in distant and stage IV PCa observed between 2011 and 2021 is similar to findings from other high-income countries.^11,16,17,18,19,20,21^ This trend implies that the more cautious recommendations issued between 2011 and 2014 in Switzerland may have led to certain shifts in PCa stage at diagnosis. This raises important concerns that some men at high risk for developing aggressive PCa are missed in current opportunistic early detection practices in Switzerland, with no organized programs currently in place. In the presence of relatively high uptake of PSA testing—more than 50% of men aged 55 to 74 years reported a prostate examination in the past 5 years from 2012 to 2022 (eFigure 10 in Supplement 1)^37,42^—this increase in advanced PCa appears in contradiction with increasing overdiagnosis. However, such observations are possible if lower-risk individuals undergo frequent testing, while higher-risk individuals are not routinely offered testing.^43^ Of note, no causality can be inferred from our study, since other factors may also explain the increases in distant and stage IV PCa, such as more sensitive imaging (particularly prostate-specific membrane antigen–positron emission tomography) or surgical techniques. Nevertheless, this study suggests that current practice in Switzerland may neither effectively identify men who could benefit from PCa screening nor mitigate overdiagnosis.^43^

Despite this, we observed a substantial decline in PCa mortality and increase in relative survival over the past decades. This aligns with prior Swiss^12,44^ and international studies^7,8^ showing similar trends. Generally, improved prognosis may be attributed both to early detection and advances in uro-oncologic treatment of PCa, which are difficult to disentangle.^4^ However, there is little correlation between mortality and incidence (the latter being more influenced by early detection practices), suggesting a more important role for advances in treatment in a context with relevant competing mortality due to other causes.^4,7^ Meanwhile, time delays between changes in early detection practices and any potential effects on incidence of metastatic PCa or PCa-related mortality also have to be considered and complicate interpretation. Relative survival in our study was highest among men aged 50 to 79 years and lowest among men younger than 50 years or 80 years or older, indicating more aggressive disease in younger patients (likely diagnosed upon symptoms) and more advanced PCa in older patients. Finally, men with localized PCa had a similar or even superior survival compared with the general population, which is implausible beyond potentially improved prevention and care for other chronic conditions. This strongly suggests that men tested for and diagnosed with PCa have a lower baseline mortality risk compared with those not tested. This may be associated with socioeconomic differences and access to health care services,^45,46^ as well as guidelines recommending testing only in men with a life expectancy of 10 years or more.^47^

### Limitations

With this study, we present a comprehensive analysis of PCa incidence, mortality and relative survival during a 42-year period, although several limitations need to be considered. First, the study is limited by the completeness of cancer registration in Switzerland. While data from few cantons were available in the early years of the study period, the increase in cantons participating in cancer registration over time also increases the confidence in the reported estimates. Due to the important change in the Swiss legislation mandating cancer registration from 2020, it may be that registration completeness has further increased in 2020 to 2021, contributing to the observed incidence increases. However, registration quality and completeness in Swiss cantons with cancer registration has been considered very high prior to this legislative change,^48^ such that we deem it unlikely that this has relevantly influenced the observed trends. Nevertheless, heterogeneity in data quality and coding practices between individual registries may still exist with respect to stage and GS information. Additionally, sociodemographic characteristics or PSA testing practices may be associated with certain registries. While all these differences may have an impact on extrapolations at the national level and on estimated trends across prognostic groups, our findings were consistent across sensitivity analyses, and we consider it unlikely that they would alter our conclusions substantially. Second, there was relevant missingness with respect to stage and GS. While we aimed to account for missing data through several methods, including multiple imputation, it may be that our assumptions were not fulfilled and our results could still be subject to residual influences of incomplete information. Third, the obtained dataset included 5-year age strata so that the individual ages of PCa cases had to be approximated, which may slightly affect relative survival estimates. Fourth, relative survival may be influenced by differences in health and sociodemographic characteristics between men diagnosed with PCa and the general population and thus represent an overestimate. Cause-specific survival would provide a lower bound for the true net survival,^49^ but could not be estimated in this study. Furthermore, improvements in relative survival may be partly subject to lead-time bias, given changing early detection practices over time. However, the extent of any such bias is challenging to estimate, given the widespread use of PSA testing since the early 2000s and concurrent advances in treatment. Fifth, the Gleason scoring system underwent several important revisions, leading to changes in grading practices over the study period.^50^ This may affect the Gleason score assigned to individual cancers and limits comparability over time. Most important modifications were made by the International Society of Urological Pathology in 2005 and in 2014. Last, due to the nature of this study, we could not empirically assess the reasons for the observed trends based on the available data. Further studies are required to establish whether the hypothesized causes hold true.

## Conclusions

In this population-based cohort study, we found indications of increasing overdiagnosis of low-risk PCa and a concurrent rise in cancers detected only after locoregional or distant metastasis in recent years in Switzerland. Meanwhile, the prognosis of PCa substantially improved over the last decades, most likely due to advances in treatment. While further studies on the causes of the observed trends are warranted and temporal relations need to be considered, these findings point to substantial potential to optimize PCa early detection by ensuring that up-to-date, evidence-based recommendations are established and consistently implemented across Switzerland.

## Notes

Supplement 1. **eTable 1.** Cancer Registration Coverage of the Male Population and Start of Cancer Registration for Different Swiss Cantons (1980-2021) **eTable 2.** Definition of SEER Stage, UICC Stage, and Gleason Score (GS) Groups Used in Subgroup Analyses **eTable 3.** Intervention Codes Used to Categorize Cases Treated With Prostatectomy **eMethods.** Measures to Account for Missing Information **eFigure 1.** Prostate Cancer Incidence by SEER Stage Based on Alternative Analyses and by Age **eFigure 2.** Prostate Cancer Incidence by UICC Stage Based on Alternative Analyses and by Age **eFigure 3.** Prostate Cancer Incidence by Gleason Score Based on Alternative Analyses and by Age **eTable 4.** Crude (Unadjusted) Incidence Rate and Directly Age-Standardized Prostate Cancer Incidence Rates (ASIRs) Based on Different Standard Populations (1980-2021) **eTable 5.** Crude (Unadjusted) Mortality Rate and Directly Age-Standardized Prostate Cancer Mortality Rates (ASMRs) Based on Different Standard Populations (1980-2021) **eFigure 4.** Flowchart of Registered Prostate Cancer Cases (1980-2021) and Inclusion in Analyses **eTable 6.** Estimated Prostate Cancer Cases and Directly Age-Standardized Prostate Cancer Incidence Rates (ASIRs), Overall and by Age Group (1980-2021) **eFigure 5.** Prostate Cancer Incidence Estimated Based on All Registries and Including Only Cancer Registries Active During the Entire Study Time Frame **eFigure 6.** Prostate Cancer Incidence by Prognostic Group After Excluding All Cases Receiving Prostate Surgery as Primary Treatment Due to Potential Upstaging (Sensitivity Analysis) **eFigure 7.** Prostate Cancer Incidence by Prognostic Group After Excluding Cancer Registries Within Their First 2 Years of Registration or With ≥30% Missingness in a Given Year (Sensitivity Analysis) **eTable 7.** Observed Prostate Cancer Deaths and Directly Age-Standardized Prostate Cancer Mortality Rates (ASMRs), Overall and by Age Group (1980-2021) **eTable 8.** Relative Survival of Prostate Cancer Cases Across Incidence Periods, Overall and by Age Group (1980-2021) **eFigure 8.** Relative Survival of Prostate Cancer Cases by Prognostic Group and Age Group (2002-2021) **eFigure 9.** Relative Survival of Prostate Cancer Cases by SEER Stage and Incidence Period (2002-2021) **eFigure 10.** External Evidence on Prostate Cancer Testing From Swiss Data Sources
eReferences

Supplement 2.
Data Sharing Statement
