# Supplementary material for: Prostate Cancer Incidence, Mortality, and Survival in Switzerland
Source: JAMA Netw Open. 2026 Apr 21;9(4):e268289. doi: 10.1001/jamanetworkopen.2026.8289 (PMC13100841; doi:10.1001/jamanetworkopen.2026.8289)
Supplement: Supplement 1. — eTable 1. Cancer Registration Coverage of the Male Population and Start of Cancer Registration for Different Swiss Cantons (1980-2021) eTable 2. Definition of SEER Stage, UICC Stage, and Gleason Score (GS) Groups Used in Subgroup Analyses eTable 3. Intervention Codes Used to Categorize Cases Treated With Prostatectomy eMethods. Measures to Account for Missing Information eFigure 1. Prostate Cancer Incidence by SEER Stage Based on Alternative Analyses and by Age eFigure 2. Prostate Cancer Incidence by UICC Stage Based on Alternative Analyses and by Age eFigure 3. Prostate Cancer Incidence by Gleason Score Based on Alternative Analyses and by Age eTable 4. Crude (Unadjusted) Incidence Rate and Directly Age-Standardized Prostate Cancer Incidence Rates (ASIRs) Based on Different Standard Populations (1980-2021) eTable 5. Crude (Unadjusted) Mortality Rate and Directly Age-Standardized Prostate Cancer Mortality Rates (ASMRs) Based on Different Standard Populations (1980-2021) eFigure 4. Flowchart of Registered Prostate Cancer Cases (1980-2021) and Inclusion in Analyses eTable 6. Estimated Prostate Cancer Cases and Directly Age-Standardized Prostate Cancer Incidence Rates (ASIRs), Overall and by Age Group (1980-2021) eFigure 5. Prostate Cancer Incidence Estimated Based on All Registries and Including Only Cancer Registries Active During the Entire Study Time Frame eFigure 6. Prostate Cancer Incidence by Prognostic Group After Excluding All Cases Receiving Prostate Surgery as Primary Treatment Due to Potential Upstaging (Sensitivity Analysis) eFigure 7. Prostate Cancer Incidence by Prognostic Group After Excluding Cancer Registries Within Their First 2 Years of Registration or With ≥30% Missingness in a Given Year (Sensitivity Analysis) eTable 7. Observed Prostate Cancer Deaths and Directly Age-Standardized Prostate Cancer Mortality Rates (ASMRs), Overall and by Age Group (1980-2021) eTable 8. Relative Survival of Prostate Cancer Cases Across Incidence Periods, Overall and [file jamanetwopen-e268289-s001.pdf]

Supplementary Online Content

Menges, D, Wildisen L, Scherer T, et al. Prostate cancer incidence, mortality and survival in Switzerland. *JAMA Netw Open*. 2026;9(4):268289. doi:10.1001/jamanetworkopen.2026.8289

eTable 1. Cancer Registration Coverage of the Male Population and Start of Cancer Registration for Different Swiss Cantons (1980-2021)

eTable 2. Definition of SEER Stage, UICC Stage, and Gleason Score (GS) Groups Used in Subgroup Analyses

eTable 3. Intervention Codes Used to Categorize Cases Treated With Prostatectomy

eMethods. Measures to Account for Missing Information

eFigure 1. Prostate Cancer Incidence by SEER Stage Based on Alternative Analyses and by Age

eFigure 2. Prostate Cancer Incidence by UICC Stage Based on Alternative Analyses and by Age

eFigure 3. Prostate Cancer Incidence by Gleason Score Based on Alternative Analyses and by Age

eTable 4. Crude (Unadjusted) Incidence Rate and Directly Age-Standardized Prostate Cancer Incidence Rates (ASIRs) Based on Different Standard Populations (1980-2021)

eTable 5. Crude (Unadjusted) Mortality Rate and Directly Age-Standardized Prostate Cancer Mortality Rates (ASMRs) Based on Different Standard Populations (1980-2021)

eFigure 4. Flowchart of Registered Prostate Cancer Cases (1980-2021) and Inclusion in Analyses

eTable 6. Estimated Prostate Cancer Cases and Directly Age-Standardized Prostate Cancer Incidence Rates (ASIRs), Overall and by Age Group (1980-2021)

eFigure 5. Prostate Cancer Incidence Estimated Based on All Registries and Including Only Cancer Registries Active During the Entire Study Time Frame

eFigure 6. Prostate Cancer Incidence by Prognostic Group After Excluding All Cases Receiving Prostate Surgery as Primary Treatment Due to Potential Upstaging (Sensitivity Analysis)

eFigure 7. Prostate Cancer Incidence by Prognostic Group After Excluding

© 2026 Menges D et al. *JAMA Network Open*.

Cancer Registries Within Their First 2 Years of Registration or With ≥30% Missingness in a Given Year (Sensitivity Analysis)

eTable 7. Observed Prostate Cancer Deaths and Directly Age-Standardized Prostate Cancer Mortality Rates (ASMRs), Overall and by Age Group (1980-2021)

eTable 8. Relative Survival of Prostate Cancer Cases Across Incidence Periods, Overall and by Age Group (1980-2021)

eFigure 8. Relative Survival of Prostate Cancer Cases by Prognostic Group and Age Group (2002-2021)

eFigure 9. Relative Survival of Prostate Cancer Cases by SEER Stage and Incidence Period (2002-2021)

eFigure 10. External Evidence on Prostate Cancer Testing From Swiss Data Sources

eReferences

This supplementary material has been provided by the authors to give readers additional information about their work.

© 2026 Menges D et al. *JAMA Network Open*.

eTable 1. Cancer Registration Coverage of the Male Population and Start of Cancer Registration for Different Swiss Cantons (1980-2021)

Year	Coverage	Cantons covered by cancer registration	Number of cantons covered
1980	41%	BS, BL, GE, VD, NE, ZH, SG, AR, AI	9/26
1985	47%	BS, BL, GE, VD, NE, ZH, SG, AR, AI	9/26
1990	53%	BS, BL, GE, VD, NE, ZH, SG, AR, AI, VS, GR	11/26
1995	53%	BS, BL, GE, VD, NE, ZH, SG, AR, AI, VS, GR, GL	12/26
2000	57%	BS, BL, GE, VD, NE, ZH, SG, AR, AI, VS, GR, GL, TI	13/26
2005	59%	BS, BL, GE, VD, NE, ZH, SG, AR, AI, VS, GR, GL, TI, JU, FR	15/26
2010	67%	BS, BL, GE, VD, NE, ZH, SG, AR, AI, VS, GR, GL, TI, JU, FR, LU, UR, OW, NW	19/26
2015	94%	BS, BL, GE, VD, NE, ZH, SG, AR, AI, VS, GR, GL, TI, JU, FR, LU, UR, OW, NW, ZG, TG, AG, BE	23/26
2020	100%	BS, BL, GE, VD, NE, ZH, SG, AR, AI, VS, GR, GL, TI, JU, FR, LU, UR, OW, NW, ZG, TG, AG, BE, SO, SZ, SH	26/26

Legend: More detailed information is available at https://nacr.ch/en/history-of-cancer-registration-in-switzerland and https://nacr.ch/en/downloads-and-links?cat=45 (last accessed 23 Feb 2026).

© 2026 Menges D et al. *JAMA Network Open*.

eTable 2. Definition of SEER Stage, UICC Stage, and Gleason Score (GS) Groups Used in Subgroup Analyses

	Subgroup	Definition	Source
SEER stage [a]	Localized	T1-2 N0 M0	1
	Regional	T3-4 N0 M0 or any T N1+ M0	
	Distant	any T any N M1	
UICC stage [a]	I	T1/2a N0 M0	2
	II	T2b/2c N0 M0	
	III	T3-4 N0 M0	
	IV	any T N1+ M0 or any T any N M1	
Gleason score [b]	≤6	GS 2-6	
	7	GS 7	
	8-10	GS 8-10	

Legend: GS = Gleason score, SEER = Surveillance, Epidemiology, and End Results, UICC = Union for International Cancer Control. [a] To determine prostate cancer stages based on SEER and UICC, we primarily used registered pathological TNM staging information. If pathological staging was not available, incomplete, or pathological TNM staging was assigned during or after systemic treatment, we used clinical TNM information for T and N status. M status was considered M1 if this was coded in clinical or pathological TNM staging, and M0 was assumed if M status was missing in the presence of T and N data. [b] GS information was used as registered. While the Gleason scoring system was revised by the International Society of Urological Pathology (ISUP) in 2005 to no longer include GS <6,[3] few cancers with GS 2-5 were still registered in subsequent years and were included in the GS ≤6 group for analysis.

© 2026 Menges D et al. *JAMA Network Open*.

eTable 3. Intervention Codes Used to Categorize Cases Treated With Prostatectomy

Code [a]	Description (German)	Description (French)	Translation (English)
Z00.R1	Chirurgische Behandlung n.n.b. (nur für Krebsregistrierung)	Chirurgie SAI (seulement pour l'enregistrement du cancer)	Surgical treamtne, not further specified (only for cancer registration)
Z00.R2	Chirurgischer Eingriff am Organ n.n.b. (nur für Krebsregistrierung)	Chirurgie : intervention sur l'organe, SAI (seulement pour l'enregistrement du cancer)	Surgical intervention on the organ, not further specified (only for cancer registration)
Z00.R4	Partielle oder totale Resektion eines Organs n.n.b. (nur für Krebsregistrierung)	Amputation partielle ou totale (seulement pour l'enregistrement du cancer)	Partial or total resection of an organ, not further specified (only for cancer registration)
Z00.R5	Radikale Operation 1 Organ n.n.b. (nur für Krebsregistrierung)	Chirurgie radicale (1 organe) (seulement pour l'enregistrement du cancer)	Radical surgery (1 organ), not further specified (only for cancer registration)
Z00.R6	Radikale Operation 2 und mehr Organe n.n.b. (nur für Krebsregistrierung)	Chirurgie radicale (2 organes ou +) (seulement pour l'enregistrement du cancer)	Radical surgery (2 organs or more), not further specified (only for cancer registration)
Z00.RD	Lokale Exzision n.n.b. (nur für Krebsregistrierung)	Excision locale sans précision (seulement pour l'enregistrement du cancer)	Local excision, not further specified (only for cancer registration)
Z00.RE	Erweiterte Exzision (nur für Krebsregistrierung)	Excision large, étendue (seulement pour l'enregistrement du cancer)	Extensive excision, not further specified (only for cancer registration)
Z00.RI	Ausgedehnter Eingriff am Tumor (nur für Krebsregistrierung)	Intervention étendue sur la tumeur (seulement pour l'enregistrement du cancer)	Extensive intervention on the tumor (only for cancer registration)
Z00.RJ	Resektion mit Entfernung unvollständig (nur für Krebsregistrierung)	Résection avec ablation incomplète (seulement pour l'enregistrement du cancer)	Resection with incomplete removal (only for cancer registration)
Z60.3	Suprapubische Prostatektomie	Prostatectomie suprapubienne	Suprapubic prostatectomy
Z60.4	Retropubische Prostatektomie	Prostatectomie rétropubienne	Retropubic prostatectomy
Z60.5X.00	Radikale Prostatektomie, n.n.bez.	Prostatectomie radicale, SAP	Radical prostatectomy, not further specified
Z60.5X.10	Radikale Prostatektomie, laparoskopisch	Prostatectomie radicale laparoscopique	Radical prostatectomy, laparoscopic
Z60.5X.20	Radikale Prostatektomie, perineal	Prostatectomie radicale périnéale	Radical prostatectomy, perineal
Z60.5X.30	Radikale Prostatektomie, retropubisch	Prostatectomie radicale rétropubienne	Radical prostatectomy, retropubic

© 2026 Menges D et al. *JAMA Network Open*.

Z60.5X.99	Radikale Prostatektomie, sonstige	Prostatectomie radicale, autre	Prostatectomie radicale, autre
Z60.61.00	Lokale Exzision einer Läsion an der Prostata, n.n.bez.	Excision locale de lésion de la prostate, SAP	Local excision of a prostatic lesion, not further specified
Z60.61.11	Lokale Exzision einer Läsion an der Prostata, suprapubisch-transvesikal	Excision locale de lésion de la prostate, abord suprapubien transvésical	Local excision of a prostatic lesion, suprapubic transvesical
Z60.61.12	Lokale Exzision einer Läsion an der Prostata, retropubisch	Excision locale de lésion de la prostate, abord rétropubien	Local excision of a prostatic lesion, retropubic
Z60.61.13	Lokale Exzision einer Läsion an der Prostata, perineal	Excision locale de lésion de la prostate, abord périnéal	Local excision of a prostatic lesion, perineal
Z60.61.99	Lokale Exzision einer Läsion an der Prostata, sonstige	Excision locale de lésion de la prostate, autre	Local excision of a prostatic lesion, other
Z60.62	Perineale Prostatektomie	Prostatectomie par voie périnéale	Perineal prostatectomy
Z60.69	Sonstige Prostatektomie, sonstige	Autre prostatectomie, autre	Other prostatectomy, other

Legend: [a] Codes correspond to Swiss CHOP codes or cancer registration codes.

© 2026 Menges D et al. *JAMA Network Open*.

eMethods. Measures to Account for Missing Information

There was relevant missing information regarding stage and Gleason scores (GS) among registered prostate cancer (PCa) cases. To account for this, we applied the following methods:

First, we restricted all prognostic subgroup analyses to data from 2002-2021, in which the proportion of cases with complete information was higher than in preceding years.

Second, we report proportions of missingness and incidence rates for cases with unknown information over time (as registered) in eFigures 1–3.

Third, we extrapolated incidence rates estimated based on cases with complete information by reducing the population at risk for each year, age group, and registry by the corresponding proportion of missingness. This analysis allows interpreting incidence trends at the population level in the presence of changes in the extent of missing data, while taking uncertainty associated with the observed sample into account. However, it assumes missingness completely at random (MCAR), which is unlikely to hold true in the context of cancer registration.

Fourth, we applied multiple imputation under a missingness at random (MAR) assumption based on the length of follow-up, status at the end of follow-up, the Nelson-Aalen estimator, incidence year, registry, and age group. This approach aimed to reduce bias due to differential completeness of staging information across registries and calendar years. For each outcome, we created 40 imputed datasets, calculated age-standardized incidence rates, and estimated pooled results using Rubin's rules to account for within- and across-imputation variation.[4] For incidence rates based on multiple imputation, we estimated 95% confidence intervals using a normal approximation of log-transformed incidence rates, allowing to pool estimated rates and corresponding variances on the log scale across imputations.[5]

In the article, reported primary analyses are based on multiple imputation. Results from 'as registered' analyses and extrapolations based on an MCAR assumption are presented in eFigures 1–3.

© 2026 Menges D et al. *JAMA Network Open*.

eFigure 1. Prostate Cancer Incidence by SEER Stage Based on Alternative Analyses and by Age

Estimated prostate cancer incidence is presented as registered (panel a), based on extrapolation of cases with missing information (panel b), and by age group based on multiple imputation (panel c), as well as proportions of cases by SEER stage and age group (panel d).

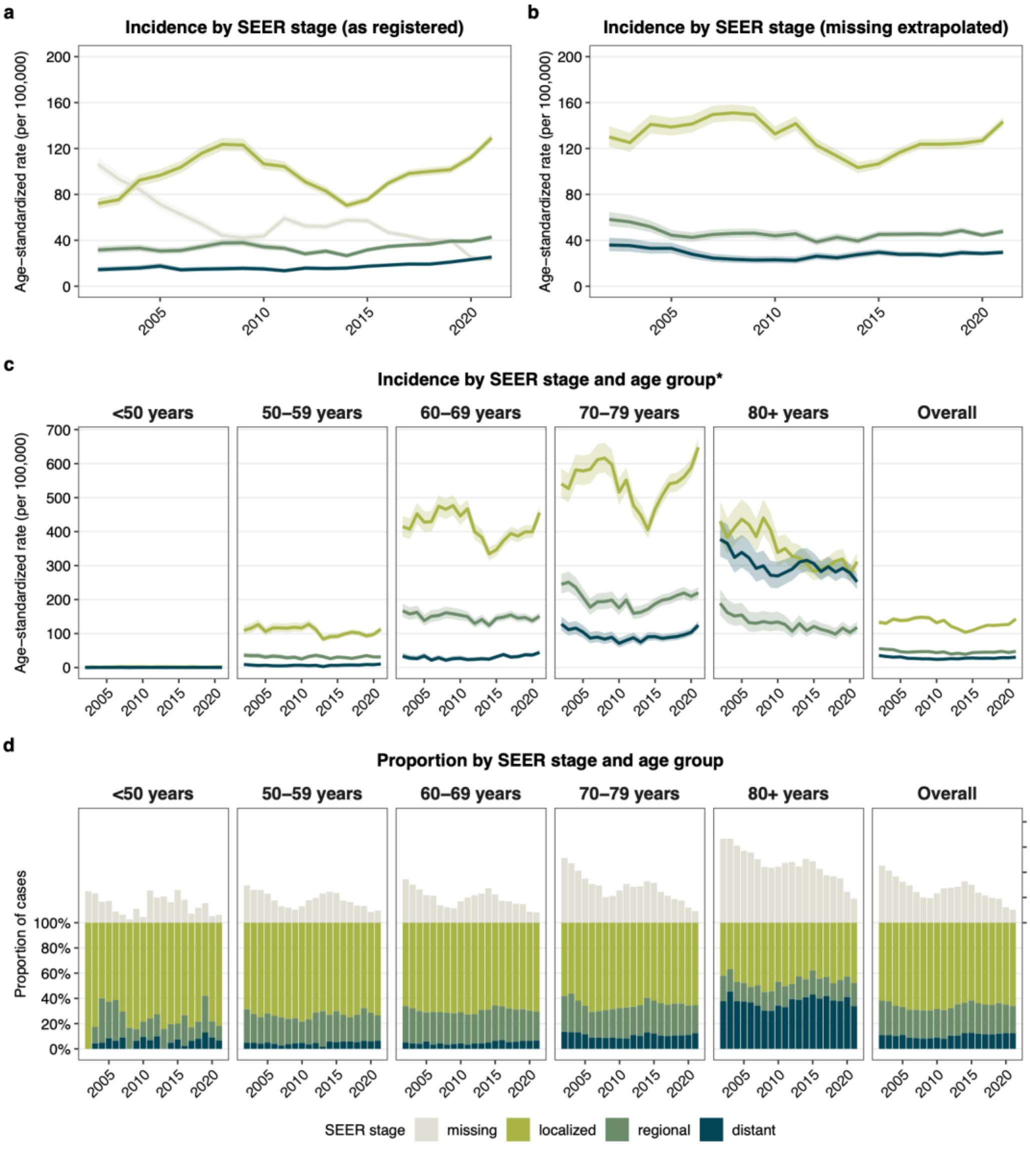

Legend: Rates per 100,000 men were directly age-standardized using the European 2013 standard population. Shaded areas represent 95% confidence intervals. Proportions of cases with missing information were estimated among all cases, proportions in subgroups were estimated among all cases without missing information. * Multiple imputation was based on length of follow-up and status at end of follow-up of cases, the Nelson-Aalen estimator, incidence year, registry, and age group.

© 2026 Menges D et al. *JAMA Network Open*.

eFigure 2. Prostate Cancer Incidence by UICC Stage Based on Alternative Analyses and by Age

Estimated prostate cancer incidence is presented as registered (panel a), based on extrapolation of cases with missing information (panel b), and by age group based on multiple imputation (panel c), as well as proportions of cases by UICC stage and age group (panel d).

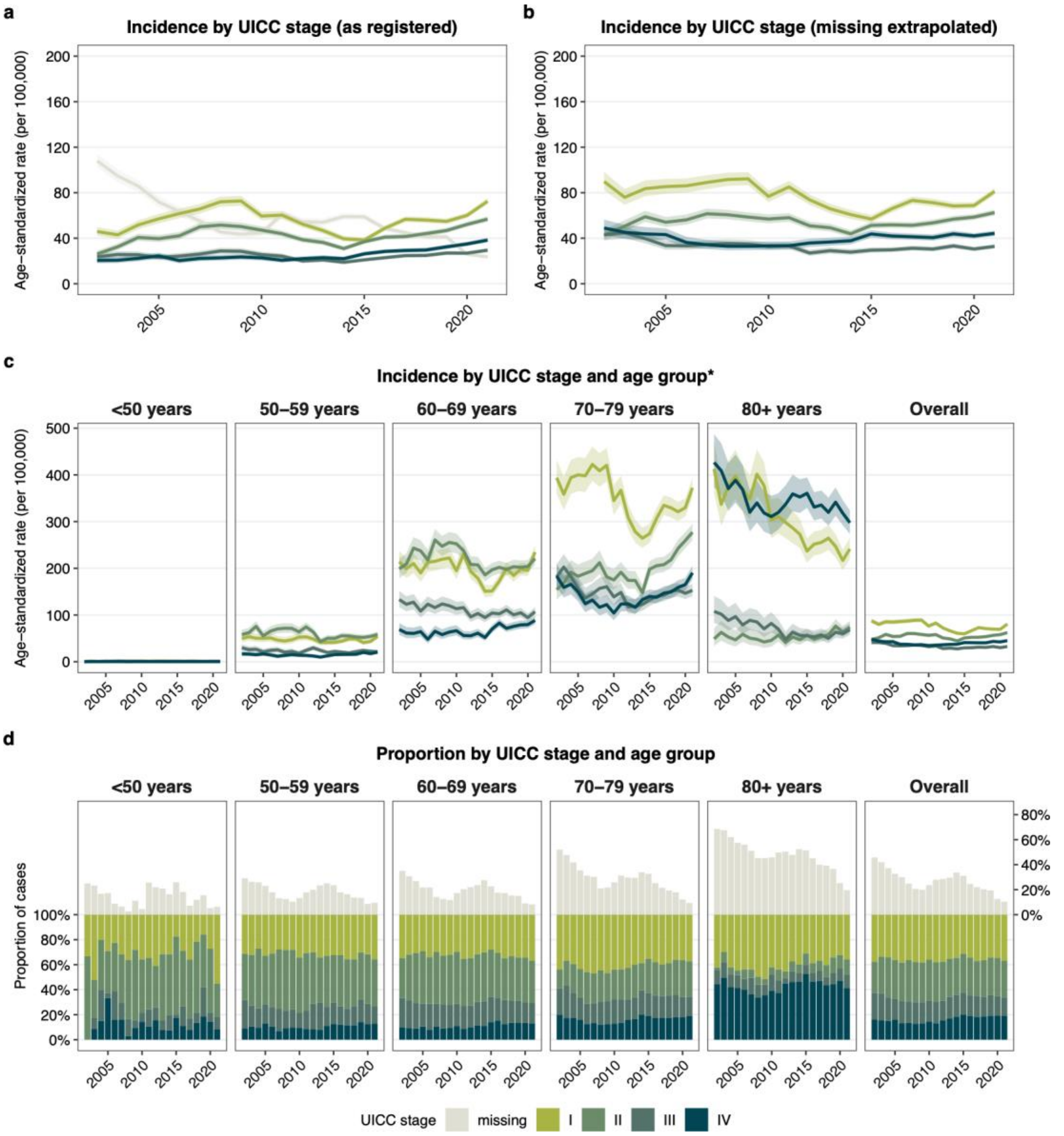

Legend: Rates per 100,000 men were directly age-standardized using the European 2013 standard population. Shaded areas represent 95% confidence intervals. Proportions of cases with missing information were estimated among all cases, proportions in subgroups were estimated among all cases without missing information. * Multiple imputation was based on length of follow-up and status at end of follow-up of cases, the Nelson-Aalen estimator, incidence year, registry, and age group.

© 2026 Menges D et al. *JAMA Network Open*.

eFigure 3. Prostate Cancer Incidence by Gleason Score Based on Alternative Analyses and by Age

Estimated prostate cancer incidence is presented as registered (panel a), based on extrapolation of cases with missing information (panel b), and by age group based on multiple imputation (panel c), as well as proportions of cases by Gleason score and age group (panel d).

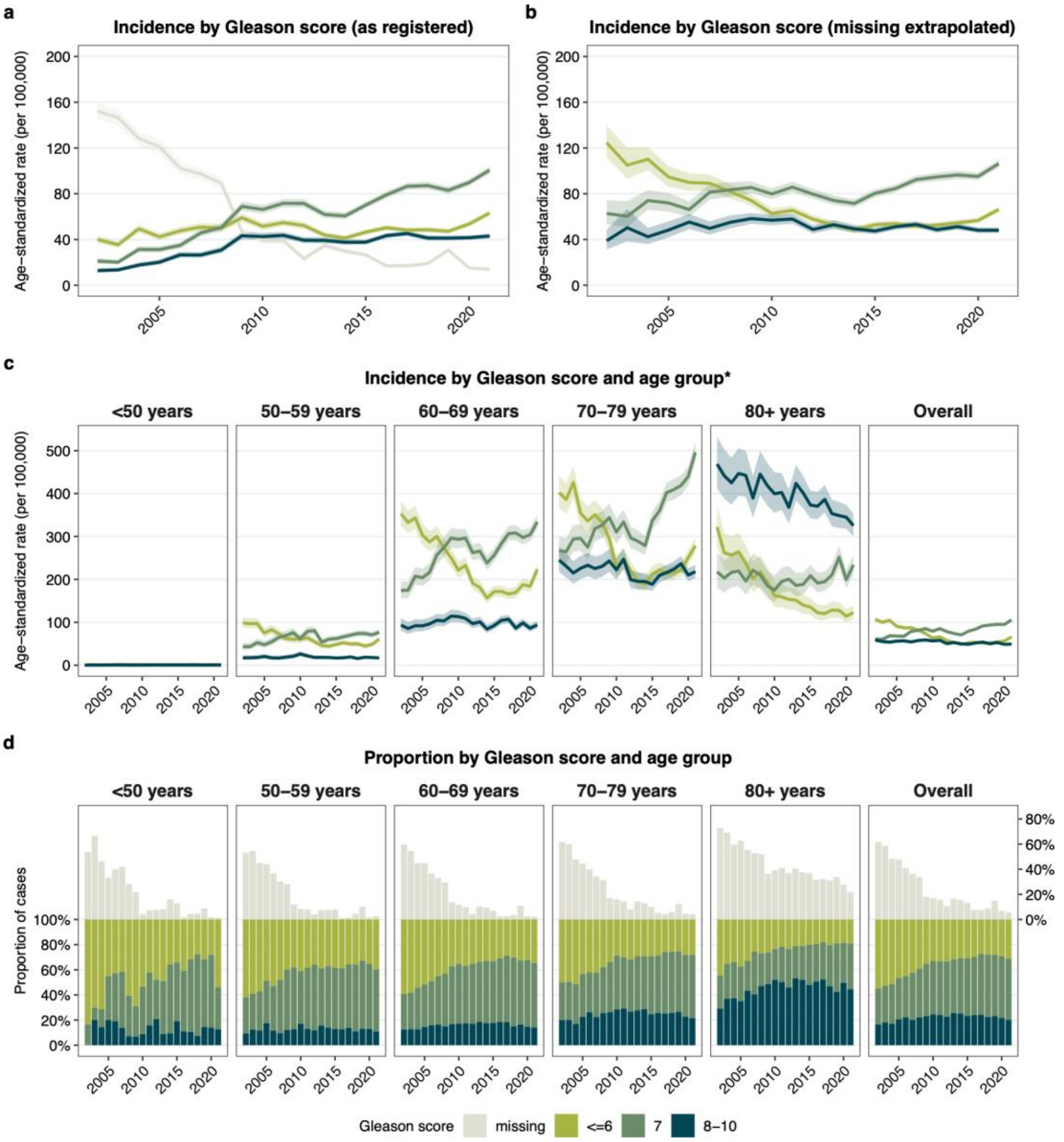

Legend: Rates per 100,000 men were directly age-standardized using the European 2013 standard population. Shaded areas represent 95% confidence intervals. Proportions of cases with missing information were estimated among all cases, proportions in subgroups were estimated among all cases without missing information. * Multiple imputation was based on length of follow-up and status at end of follow-up of cases, the Nelson-Aalen estimator, incidence year, registry, and age group.

© 2026 Menges D et al. *JAMA Network Open*.

eTable 4. Crude (Unadjusted) Incidence Rate and Directly Age-Standardized Prostate Cancer Incidence Rates (ASIRs) Based on Different Standard Populations (1980-2021)

Incidence year	Unadjusted	European 2013 [a]	European 1976 [b]	WHO World 2000-2025 (SEER correction) [c,d]
	Crude rate (95% CI)	ASIR (95% CI)	ASIR (95% CI)	ASIR (95% CI)
1980	68.6	125.6 (116.4 to 135.6)	67.8 (63.1 to 72.9)	51.2 (47.7 to 54.9)
1981	73.4	135.4 (126.7 to 144.8)	73.2 (68.8 to 78.0)	54.9 (51.6 to 58.4)
1982	71.8	130.7 (122.2 to 139.9)	70.9 (66.5 to 75.6)	53.4 (50.2 to 56.9)
1983	73.3	134.3 (125.6 to 143.5)	72.2 (67.8 to 76.9)	54.1 (50.8 to 57.5)
1984	75.1	137.2 (128.6 to 146.5)	72.5 (68.1 to 77.2)	54.4 (51.1 to 57.8)
1985	86.4	154.3 (145.3 to 163.9)	82.5 (77.9 to 87.4)	62.0 (58.5 to 65.6)
1986	86.1	151.2 (142.4 to 160.5)	81.5 (77.0 to 86.3)	61.3 (57.9 to 64.8)
1987	92.7	158.9 (150.1 to 168.2)	86.4 (81.7 to 91.3)	65.0 (61.6 to 68.7)
1988	92.0	156.4 (147.9 to 165.5)	85.0 (80.5 to 89.8)	64.0 (60.6 to 67.6)
1989	97.3	164.0 (155.7 to 172.7)	90.0 (85.5 to 94.6)	67.7 (64.4 to 71.2)
1990	94.1	157.8 (149.8 to 166.3)	87.2 (82.8 to 91.7)	65.5 (62.3 to 68.9)
1991	101.9	170.5 (162.2 to 179.1)	94.7 (90.2 to 99.4)	71.1 (67.7 to 74.6)
1992	106.6	176.9 (168.6 to 185.5)	98.5 (94.0 to 103.2)	74.3 (70.9 to 77.9)
1993	107.6	176.4 (168.3 to 184.8)	99.0 (94.5 to 103.6)	74.8 (71.4 to 78.3)
1994	113.6	185.7 (177.5 to 194.3)	104.4 (99.8 to 109.2)	78.6 (75.2 to 82.2)
1995	117.2	188.3 (180.1 to 196.8)	108.0 (103.4 to 112.8)	81.4 (77.9 to 85.0)
1996	119.2	189.9 (181.9 to 198.3)	108.7 (104.2 to 113.4)	81.8 (78.4 to 85.4)
1997	126.1	196.9 (188.8 to 205.3)	114.0 (109.4 to 118.8)	85.8 (82.4 to 89.4)
1998	136.4	207.7 (199.5 to 216.2)	122.7 (117.9 to 127.6)	92.5 (88.9 to 96.2)
1999	136.0	204.2 (196.2 to 212.5)	120.8 (116.2 to 125.7)	91.1 (87.6 to 94.8)
2000	141.7	208.2 (200.2 to 216.5)	125.9 (121.1 to 130.9)	94.6 (91.0 to 98.3)

© 2026 Menges D et al. *JAMA Network Open*.

Incidence year	Unadjusted	European 2013 [a]	European 1976 [b]	WHO World 2000-2025 (SEER correction) [c,d]
	Crude rate (95% CI)	ASIR (95% CI)	ASIR (95% CI)	ASIR (95% CI)
2001	153.4	218.5 (210.5 to 226.8)	135.2 (130.3 to 140.3)	101.8 (98.1 to 105.6)
2002	160.3	223.6 (215.6 to 231.8)	140.1 (135.1 to 145.2)	105.3 (101.6 to 109.2)
2003	157.4	215.5 (207.8 to 223.6)	136.4 (131.5 to 141.4)	102.8 (99.1 to 106.6)
2004	168.6	226.6 (218.8 to 234.7)	144.4 (139.5 to 149.5)	108.8 (105.1 to 112.6)
2005	161.4	214.8 (207.3 to 222.6)	135.5 (130.8 to 140.4)	102.1 (98.5 to 105.8)
2006	162.3	211.3 (203.9 to 218.8)	134.6 (130.0 to 139.4)	101.7 (98.2 to 105.3)
2007	172.4	220.2 (212.9 to 227.8)	141.7 (137.0 to 146.5)	107.1 (103.5 to 110.8)
2008	173.9	220.3 (213.0 to 227.9)	140.9 (136.3 to 145.7)	106.3 (102.8 to 110.0)
2009	174.6	217.9 (210.7 to 225.2)	140.7 (136.1 to 145.4)	105.9 (102.5 to 109.5)
2010	164.0	201.3 (194.9 to 207.9)	130.3 (126.1 to 134.5)	98.2 (95.1 to 101.4)
2011	172.4	209.2 (202.9 to 215.6)	135.8 (131.8 to 140.0)	102.3 (99.3 to 105.5)
2012	154.5	186.1 (180.5 to 191.9)	120.5 (116.9 to 124.3)	90.9 (88.2 to 93.8)
2013	152.0	181.7 (176.5 to 187.0)	116.4 (113.1 to 119.8)	87.7 (85.2 to 90.3)
2014	146.3	173.5 (168.9 to 178.2)	110.6 (107.7 to 113.6)	83.3 (81.1 to 85.6)
2015	154.8	180.7 (176.1 to 185.4)	115.4 (112.4 to 118.4)	87.0 (84.8 to 89.3)
2016	164.7	189.5 (184.8 to 194.3)	121.8 (118.8 to 124.9)	91.7 (89.4 to 94.1)
2017	173.6	197.0 (192.3 to 201.8)	127.2 (124.1 to 130.3)	95.8 (93.5 to 98.2)
2018	175.5	196.9 (192.2 to 201.6)	126.0 (123.0 to 129.1)	94.8 (92.6 to 97.2)
2019	182.8	202.6 (198.0 to 207.2)	129.4 (126.5 to 132.4)	97.4 (95.1 to 99.7)
2020	183.0	199.9 (195.4 to 204.4)	128.2 (125.3 to 131.1)	96.5 (94.4 to 98.8)
2021	204.7	220.6 (216.0 to 225.3)	142.3 (139.3 to 145.4)	107.4 (105.2 to 109.8)

Legend: ASIR = age-standardized incidence rate, CI = confidence interval, SEER = Surveillance, Epidemiology, and End Results, WHO = World Health Organization. [a] See European Commission 2013, *Revision of the European Standard Population: 2013 edition* (https://data.europa.eu/doi/ 10.2785/11470, last accessed 2 Dec 2024). [b] See Waterhouse et al., IARC 1976, *Standard population (World and European)*. [c] See Ahmad et al. 2001, *Age standardization of rates: a new WHO standard. Geneva: World Health Organization* 2001. [d] See SEER Program 2025, https://seer.cancer.gov/ stdpopulations/world.who.html (last accessed 20 Jan 2025).

© 2026 Menges D et al. *JAMA Network Open*.

eTable 5. Crude (Unadjusted) Mortality Rate and Directly Age-Standardized Prostate Cancer Mortality Rates (ASMRs) Based on Different Standard Populations (1980-2021)

Incidence year	Unadjusted	European 2013 [a]	European 1976 [b]	WHO World 2000-2025 (SEER correction) [c,d]
	Crude rate (95% CI)	ASMR (95% CI)	ASMR (95% CI)	ASMR (95% CI)
1980	30.8	64.9 (60.5 to 69.7)	31.9 (29.8 to 34.1)	23.8 (22.2 to 25.4)
1981	37.7	81.0 (76.0 to 86.4)	39.1 (36.8 to 41.5)	29.1 (27.5 to 30.9)
1982	37.1	77.5 (72.7 to 82.6)	37.5 (35.3 to 39.8)	28.2 (26.5 to 29.9)
1983	35.9	73.8 (69.2 to 78.7)	35.9 (33.8 to 38.2)	26.9 (25.3 to 28.5)
1984	37.5	75.5 (70.9 to 80.4)	36.8 (34.6 to 39.0)	27.5 (25.9 to 29.2)
1985	42.0	84.6 (79.7 to 89.7)	41.0 (38.7 to 43.3)	30.4 (28.8 to 32.1)
1986	39.8	78.1 (73.6 to 82.9)	38.4 (36.2 to 40.6)	28.5 (26.9 to 30.2)
1987	39.8	77.9 (73.4 to 82.6)	37.5 (35.4 to 39.6)	27.8 (26.3 to 29.4)
1988	42.3	80.7 (76.2 to 85.4)	39.2 (37.1 to 41.4)	29.1 (27.6 to 30.7)
1989	42.5	82.2 (77.7 to 86.9)	39.3 (37.2 to 41.5)	29.0 (27.5 to 30.6)
1990	45.0	85.2 (80.8 to 89.9)	40.7 (38.6 to 42.9)	30.3 (28.8 to 31.9)
1991	43.2	80.8 (76.5 to 85.3)	38.8 (36.8 to 41.0)	29.1 (27.6 to 30.6)
1992	43.2	80.9 (76.7 to 85.4)	39.2 (37.1 to 41.3)	29.0 (27.5 to 30.6)
1993	44.2	82.6 (78.3 to 87.0)	39.7 (37.7 to 41.8)	29.4 (27.9 to 31.0)
1994	45.0	83.7 (79.4 to 88.1)	40.1 (38.1 to 42.2)	29.6 (28.1 to 31.2)
1995	37.8	68.7 (64.9 to 72.6)	33.4 (31.6 to 35.3)	24.7 (23.4 to 26.1)
1996	37.2	67.0 (63.3 to 70.9)	32.2 (30.5 to 34.1)	23.9 (22.6 to 25.3)
1997	36.7	65.1 (61.5 to 68.9)	31.5 (29.8 to 33.3)	23.4 (22.1 to 24.7)
1998	36.4	63.3 (59.8 to 67.0)	30.7 (29.0 to 32.4)	22.9 (21.6 to 24.2)
1999	38.9	67.1 (63.5 to 70.8)	32.5 (30.7 to 34.3)	24.2 (22.9 to 25.5)
2000	37.2	63.6 (60.1 to 67.2)	30.7 (29.0 to 32.4)	22.8 (21.5 to 24.1)
2001	37.3	62.2 (58.8 to 65.7)	30.1 (28.5 to 31.8)	22.3 (21.1 to 23.5)
2002	35.6	58.5 (55.3 to 61.9)	28.1 (26.6 to 29.7)	20.8 (19.7 to 22.0)
2003	36.7	59.9 (56.6 to 63.3)	28.6 (27.0 to 30.2)	21.1 (20.0 to 22.3)
2004	36.1	58.0 (54.8 to 61.3)	27.8 (26.3 to 29.4)	20.5 (19.4 to 21.7)
2005	34.7	54.6 (51.6 to 57.8)	25.9 (24.5 to 27.4)	19.2 (18.2 to 20.3)
2006	34.9	53.6 (50.7 to 56.7)	25.6 (24.2 to 27.1)	19.0 (17.9 to 20.1)
2007	34.1	51.5 (48.6 to 54.4)	24.5 (23.1 to 25.9)	18.1 (17.1 to 19.1)
2008	34.9	52.4 (49.6 to 55.4)	24.8 (23.5 to 26.2)	18.2 (17.3 to 19.3)
2009	33.5	49.1 (46.4 to 51.9)	23.2 (22.0 to 24.6)	17.2 (16.2 to 18.2)
2010	36.8	53.4 (50.7 to 56.3)	25.0 (23.7 to 26.3)	18.4 (17.4 to 19.4)
2011	35.0	50.3 (47.6 to 53.1)	23.7 (22.4 to 25.0)	17.4 (16.4 to 18.3)
2012	32.3	45.9 (43.4 to 48.5)	21.7 (20.5 to 22.9)	15.9 (15.0 to 16.8)
2013	33.8	47.3 (44.8 to 49.9)	22.3 (21.1 to 23.5)	16.4 (15.5 to 17.3)
2014	32.9	45.2 (42.8 to 47.7)	21.2 (20.1 to 22.4)	15.5 (14.7 to 16.4)

© 2026 Menges D et al. *JAMA Network Open*.

Incidence year	Unadjusted	European 2013 [a]	European 1976 [b]	WHO World 2000-2025 (SEER correction) [c,d]
	Crude rate (95% CI)	ASMR (95% CI)	ASMR (95% CI)	ASMR (95% CI)
2015	33.1	44.6 (42.3 to 47.1)	21.0 (19.9 to 22.2)	15.4 (14.6 to 16.2)
2016	30.3	40.1 (37.9 to 42.4)	18.9 (17.9 to 20.0)	13.9 (13.1 to 14.7)
2017	33.9	44.2 (41.9 to 46.5)	20.6 (19.6 to 21.8)	15.0 (14.2 to 15.8)
2018	33.1	42.0 (39.8 to 44.3)	19.6 (18.6 to 20.7)	14.3 (13.5 to 15.1)
2019	32.4	39.9 (37.8 to 42.1)	18.9 (17.9 to 19.9)	13.8 (13.0 to 14.5)
2020	30.9	37.5 (35.5 to 39.6)	17.5 (16.6 to 18.5)	12.8 (12.1 to 13.5)
2021	29.1	34.8 (32.9 to 36.8)	16.4 (15.5 to 17.3)	11.9 (11.2 to 12.6)

Legend: ASMR = age-standardized mortality rate, CI = confidence interval, SEER = Surveillance, Epidemiology, and End Results, WHO = World Health Organization. [a] See European Commission 2013, *Revision of the European Standard Population: 2013 edition* (https://data.europa.eu/doi/ 10.2785/11470, last accessed 2 Dec 2024). [b] See Waterhouse et al., IARC 1976, *Standard population (World and European)*. [c] See Ahmad et al. 2001, *Age standardization of rates: a new WHO standard. Geneva: World Health Organization* 2001. [d] See SEER Program 2025, https://seer.cancer.gov/stdpopulations/world.who.html (last accessed 20 Jan 2025).

© 2026 Menges D et al. *JAMA Network Open*.

eFigure 4. Flowchart of Registered Prostate Cancer Cases (1980-2021) and Inclusion in Analyses

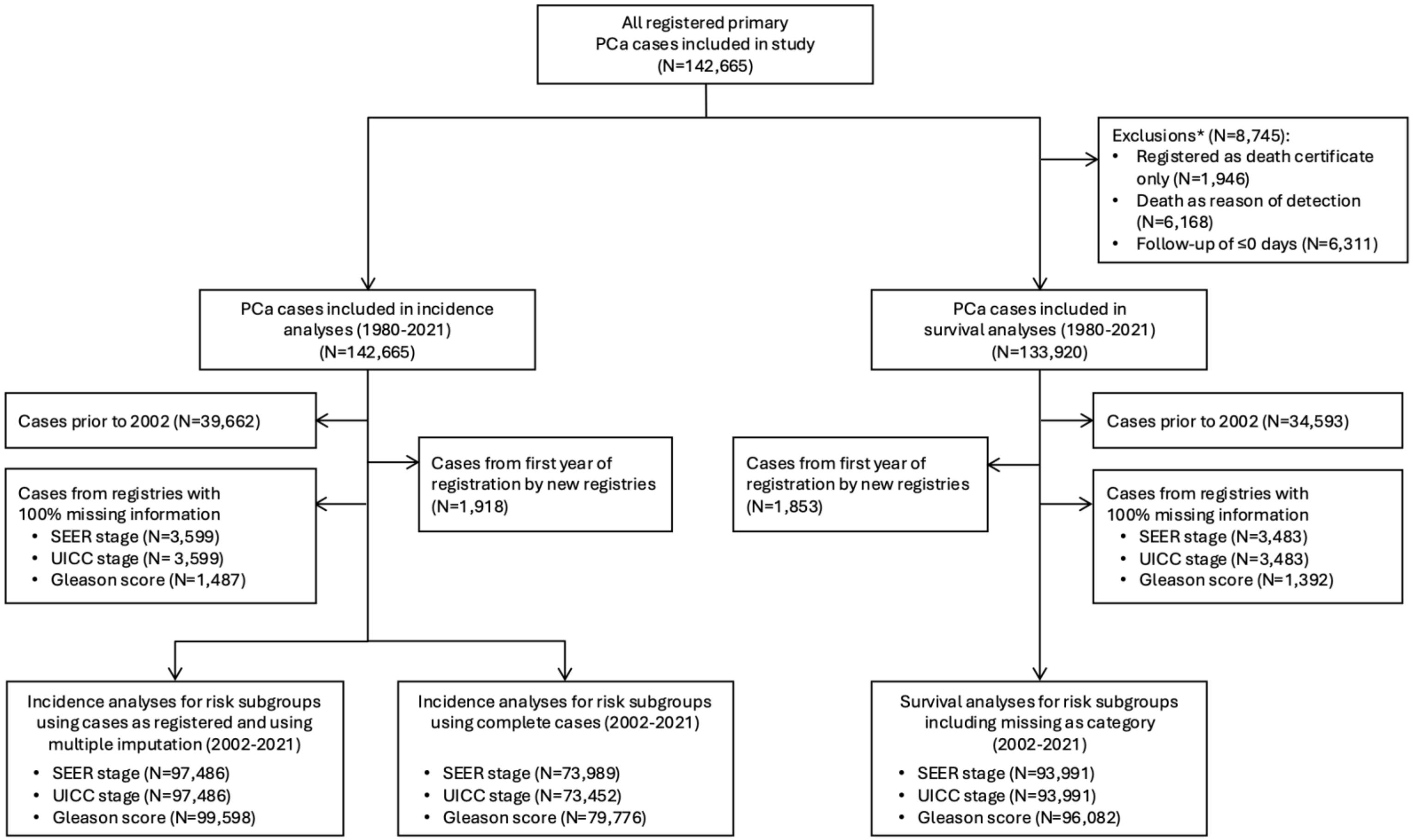

Legend: PCa = prostate cancer, NACR = National Agency for Cancer Registration, SEER = Surveillance, Epidemiology, and End Results, UICC = Union for International Cancer Control.
* Overlaps in the reasons for exclusion were possible.

© 2026 Menges D et al. *JAMA Network Open*.

eTable 6. Estimated Prostate Cancer Cases and Directly Age-Standardized Prostate Cancer Incidence Rates (ASIRs), Overall and by Age Group (1980-2021)

Incidence year	<50 years		50-59 years		60-69 years		70-79 years		80+ years		Overall	
	Est. cases	ASIR (95% CI)	Est. cases	ASIR (95% CI)	Est. cases	ASIR (95% CI)	Est. cases	ASIR (95% CI)	Est. cases	ASIR (95% CI)	Est. cases	ASIR (95% CI)
1980	8	0.3 (0.1 to 1.0)	94	27.9 (19.5 to 39.0)	497	192.2 (165.3 to 222.3)	956	557.9 (502.2 to 618.6)	540	986.3 (851.4 to 1139.9)	2,094	125.6 (116.4 to 135.6)
1981	18	0.8 (0.3 to 1.6)	105	31.1 (22.9 to 41.2)	531	205.1 (180.0 to 232.8)	1,064	619.4 (565.7 to 677.1)	539	1028.1 (899.0 to 1172.5)	2,257	135.4 (126.7 to 144.8)
1982	6	0.3 (0.1 to 0.8)	100	29.2 (21.5 to 38.8)	549	212.0 (186.3 to 240.3)	998	571.4 (520.1 to 626.5)	581	1016.5 (891.3 to 1156.6)	2,234	130.7 (122.2 to 139.9)
1983	2	0.1 (0.0 to 0.6)	114	33.3 (25.1 to 43.4)	525	203.3 (178.0 to 231.3)	1,043	592.2 (540.1 to 648.1)	608	1060.9 (932.3 to 1204.2)	2,292	134.3 (125.6 to 143.5)
1984	8	0.4 (0.1 to 1.0)	79	22.9 (16.3 to 31.5)	464	182.0 (157.9 to 208.9)	1,134	630.9 (577.5 to 688.0)	671	1124.2 (995.0 to 1267.3)	2,356	137.2 (128.6 to 146.5)
1985	7	0.3 (0.1 to 0.9)	111	31.6 (23.5 to 41.5)	576	223.0 (196.5 to 252.2)	1,233	680.3 (625.1 to 739.2)	797	1259.9 (1127.4 to 1405.4)	2,723	154.3 (145.3 to 163.9)
1986	19	0.8 (0.4 to 1.6)	108	30.5 (22.7 to 40.1)	587	222.4 (196.3 to 251.0)	1,245	688.3 (632.8 to 747.6)	771	1180.7 (1054.3 to 1319.5)	2,731	151.2 (142.4 to 160.5)
1987	9	0.4 (0.1 to 1.0)	129	36.0 (27.6 to 46.3)	670	249.4 (221.9 to 279.5)	1,330	738.0 (680.1 to 799.6)	819	1174.2 (1053.4 to 1306.7)	2,957	158.9 (150.1 to 168.2)
1988	7	0.3 (0.1 to 0.9)	126	34.6 (26.4 to 44.6)	664	241.8 (215.3 to 270.9)	1,329	740.8 (682.2 to 803.1)	832	1142.0 (1026.7 to 1268.2)	2,958	156.4 (147.9 to 165.5)
1989	6	0.2 (0.0 to 0.8)	165	44.9 (35.8 to 55.7)	746	266.3 (240.3 to 294.5)	1,324	741.2 (685.6 to 800.3)	916	1208.6 (1096.0 to 1330.9)	3,156	164.0 (155.7 to 172.7)

© 2026 Menges D et al. *JAMA Network Open*.

Incidence year	<50 years		50-59 years		60-69 years		70-79 years		80+ years		Overall	
	**Est. cases**	**ASIR (95% CI)**	**Est. cases**	**ASIR (95% CI)**	**Est. cases**	**ASIR (95% CI)**	**Est. cases**	**ASIR (95% CI)**	**Est. cases**	**ASIR (95% CI)**	**Est. cases**	**ASIR (95% CI)**
1990	17	0.7 (0.3 to 1.4)	137	36.9 (28.9 to 46.7)	746	263.5 (237.6 to 291.4)	1,367	761.9 (705.5 to 821.7)	817	1070.2 (965.4 to 1184.4)	3,085	157.8 (149.8 to 166.3)
1991	6	0.2 (0.0 to 0.7)	170	45.3 (36.3 to 55.9)	825	291.9 (264.5 to 321.4)	1,489	822.8 (764.5 to 884.4)	892	1131.5 (1025.7 to 1246.3)	3,383	170.5 (162.2 to 179.1)
1992	21	0.9 (0.4 to 1.6)	161	42.5 (33.9 to 52.7)	920	324.7 (296.1 to 355.5)	1,487	816.3 (758.6 to 877.4)	990	1195.8 (1091.0 to 1308.8)	3,579	176.9 (168.6 to 185.5)
1993	16	0.7 (0.3 to 1.3)	175	45.2 (36.5 to 55.6)	912	320.2 (292.0 to 350.4)	1,537	832.3 (774.7 to 893.3)	1,007	1163.1 (1062.7 to 1271.4)	3,646	176.4 (168.3 to 184.8)
1994	11	0.4 (0.1 to 1.0)	206	53.2 (43.7 to 64.1)	994	346.1 (316.8 to 377.4)	1,587	852.3 (794.2 to 913.8)	1,082	1234.9 (1132.7 to 1344.7)	3,880	185.7 (177.5 to 194.3)
1995	27	1.1 (0.6 to 1.8)	264	66.7 (56.1 to 78.8)	1,092	374.4 (344.3 to 406.6)	1,630	866.4 (808.4 to 927.6)	1,016	1151.0 (1052.5 to 1257.0)	4,028	188.3 (180.1 to 196.8)
1996	19	0.8 (0.4 to 1.4)	265	65.7 (55.6 to 77.3)	1,129	383.3 (353.6 to 415.0)	1,672	872.4 (815.3 to 932.7)	1,031	1159.6 (1062.8 to 1263.5)	4,116	189.9 (181.9 to 198.3)
1997	21	0.9 (0.4 to 1.6)	320	76.8 (65.9 to 89.2)	1,213	406.8 (376.3 to 439.2)	1,759	895.9 (839.2 to 955.6)	1,052	1171.7 (1074.8 to 1275.8)	4,364	196.9 (188.8 to 205.3)
1998	22	0.9 (0.4 to 1.6)	387	89.9 (78.3 to 102.9)	1,458	482.0 (449.0 to 516.8)	1,825	911.0 (854.1 to 971.0)	1,045	1151.4 (1056.1 to 1253.8)	4,737	207.7 (199.5 to 216.2)
1999	17	0.7 (0.3 to 1.3)	399	90.0 (78.5 to 102.6)	1,465	478.1 (445.5 to 512.5)	1,833	895.2 (839.9 to 953.4)	1,033	1121.6 (1027.8 to 1222.2)	4,746	204.2 (196.2 to 212.5)

© 2026 Menges D et al. *JAMA Network Open*.

Incidence year	<50 years		50-59 years		60-69 years		70-79 years		80+ years		Overall	
	Est. cases	ASIR (95% CI)	Est. cases	ASIR (95% CI)	Est. cases	ASIR (95% CI)	Est. cases	ASIR (95% CI)	Est. cases	ASIR (95% CI)	Est. cases	ASIR (95% CI)
2000	32	1.3 (0.8 to 2.1)	540	117.9 (104.8 to 132.2)	1,526	490.3 (457.3 to 525.1)	1,883	903.4 (848.0 to 961.7)	994	1075.3 (984.9 to 1172.5)	4,975	208.2 (200.2 to 216.5)
2001	35	1.4 (0.9 to 2.2)	599	127.6 (114.2 to 142.2)	1,840	582.9 (547.5 to 620.1)	1,975	934.5 (878.8 to 992.9)	968	985.6 (901.5 to 1076.2)	5,417	218.5 (210.5 to 226.8)
2002	25	1.0 (0.5 to 1.8)	742	155.2 (140.4 to 171.1)	1,972	613.1 (576.8 to 651.2)	1,948	908.4 (854.0 to 965.5)	1,019	994.5 (910.8 to 1084.3)	5,706	223.6 (215.6 to 231.8)
2003	58	2.4 (1.6 to 3.4)	762	157.1 (142.3 to 173.2)	1,959	592.9 (558.0 to 629.7)	1,918	881.8 (828.4 to 938.0)	949	906.8 (828.5 to 991.3)	5,646	215.5 (207.8 to 223.6)
2004	42	1.7 (1.1 to 2.6)	817	167.6 (152.3 to 184.0)	2,181	642.7 (606.6 to 680.5)	2,072	934.5 (880.5 to 991.2)	983	898.0 (821.6 to 980.4)	6,096	226.6 (218.8 to 234.7)
2005	51	2.1 (1.4 to 3.0)	702	143.0 (128.8 to 158.4)	2,108	598.7 (564.4 to 634.6)	1,979	875.8 (824.1 to 930.0)	1,036	931.1 (854.1 to 1014.0)	5,877	214.8 (207.3 to 222.6)
2006	63	2.6 (1.8 to 3.6)	759	153.9 (139.5 to 169.6)	2,160	599.4 (565.7 to 634.8)	1,956	849.0 (798.8 to 901.7)	1,013	871.2 (799.2 to 948.7)	5,951	211.3 (203.9 to 218.8)
2007	85	3.5 (2.5 to 4.6)	767	155.0 (140.5 to 170.6)	2,465	661.5 (626.5 to 698.0)	2,096	894.2 (843.0 to 947.9)	971	813.1 (745.0 to 886.5)	6,384	220.2 (212.9 to 227.8)
2008	65	2.6 (1.8 to 3.6)	748	149.6 (135.4 to 165.0)	2,519	650.3 (616.0 to 686.1)	2,128	889.9 (839.0 to 943.2)	1,074	873.2 (803.3 to 948.2)	6,533	220.3 (213.0 to 227.9)
2009	61	2.4 (1.7 to 3.4)	794	156.9 (142.4 to 172.5)	2,637	660.7 (626.8 to 696.1)	2,152	881.6 (832.0 to 933.5)	1,006	797.6 (731.5 to 868.5)	6,649	217.9 (210.7 to 225.2)
2010	70	2.8 (2.0 to 3.7)	765	148.9 (135.6 to 163.2)	2,575	627.6 (596.7 to 659.7)	1,908	767.5 (724.3 to 812.7)	996	766.1 (705.6 to 830.7)	6,314	201.3 (194.9 to 207.9)
2011	54	2.1 (1.5 to 2.9)	866	164.4 (151.0 to 178.7)	2,683	638.1 (608.3 to 669.1)	2,100	827.3 (784.2 to 872.3)	1,015	756.7 (699.3 to 817.8)	6,718	209.2 (202.9 to 215.6)
2012	69	2.7 (2.0 to 3.6)	833	153.4 (141.1 to 166.4)	2,342	550.1 (523.5 to 577.8)	1,870	715.7 (677.3 to 755.7)	981	722.0 (667.8 to 779.7)	6,094	186.1 (180.5 to 191.9)
2013	63	2.5 (1.8 to 3.2)	695	124.4 (114.3 to 135.3)	2,385	553.3 (528.4 to 579.1)	1,852	681.7 (647.1 to 717.7)	1,079	768.2 (716.3 to 823.1)	6,074	181.7 (176.5 to 187.0)

© 2026 Menges D et al. *JAMA Network Open*.

Incidence year	<50 years		50-59 years		60-69 years		70-79 years		80+ years		Overall	
	**Est. cases**	**ASIR (95% CI)**	**Est. cases**	**ASIR (95% CI)**	**Est. cases**	**ASIR (95% CI)**	**Est. cases**	**ASIR (95% CI)**	**Est. cases**	**ASIR (95% CI)**	**Est. cases**	**ASIR (95% CI)**
2014	60	2.3 (1.7 to 3.0)	737	128.0 (118.6 to 138.0)	2,143	492.5 (471.1 to 514.6)	1,918	679.9 (648.8 to 712.2)	1,066	739.6 (694.0 to 787.6)	5,924	173.5 (168.9 to 178.2)
2015	58	2.2 (1.7 to 2.9)	756	127.2 (118.0 to 136.9)	2,284	520.5 (498.6 to 543.1)	2,165	738.3 (706.4 to 771.3)	1,082	717.4 (673.5 to 763.6)	6,345	180.7 (176.1 to 185.4)
2016	58	2.2 (1.7 to 2.9)	829	135.7 (126.3 to 145.6)	2,475	560.0 (537.3 to 583.3)	2,408	791.2 (758.7 to 824.7)	1,061	684.3 (642.1 to 728.7)	6,831	189.5 (184.8 to 194.3)
2017	73	2.8 (2.2 to 3.5)	864	138.6 (129.2 to 148.5)	2,589	579.8 (556.9 to 603.5)	2,625	831.9 (799.3 to 865.6)	1,122	701.5 (659.4 to 745.7)	7,274	197.0 (192.3 to 201.8)
2018	50	1.9 (1.4 to 2.5)	868	136.5 (127.2 to 146.2)	2,556	568.6 (546.0 to 591.9)	2,774	854.9 (822.2 to 888.5)	1,160	699.7 (658.4 to 743.0)	7,408	196.9 (192.2 to 201.6)
2019	48	1.8 (1.3 to 2.4)	890	137.0 (128.0 to 146.5)	2,699	593.0 (570.5 to 616.2)	2,901	872.9 (840.9 to 905.8)	1,237	724.2 (683.6 to 766.6)	7,775	202.6 (198.0 to 207.2)
2020	58	2.2 (1.7 to 2.8)	906	137.2 (128.4 to 146.4)	2,662	574.0 (552.4 to 596.3)	3,065	900.9 (869.3 to 933.4)	1,153	658.3 (620.7 to 697.7)	7,844	199.9 (195.4 to 204.4)
2021	64	2.4 (1.9 to 3.1)	1,029	154.5 (145.2 to 164.3)	3,076	651.2 (628.3 to 674.7)	3,433	992.7 (959.8 to 1026.5)	1,244	681.5 (644.0 to 720.7)	8,846	220.6 (216.0 to 225.3)

Legend: ASIR = age-standardized incidence rate, CI = confidence interval, est. cases = estimated cases. The total number of prostate cancer cases in Switzerland was estimated based on an extrapolation of the observed cases within strata of age group, language region, and incidence year, due to incomplete cancer registration coverage of the country between 1980-2019.

© 2026 Menges D et al. *JAMA Network Open*.

eFigure 5. Prostate Cancer Incidence Estimated Based on All Registries and Including Only Cancer Registries Active During the Entire Study Time Frame

Dark lines represent prostate cancer incidence estimated based on all registries (primary analysis), while light lines represent incidence estimated using data from registries active over the entire study timeframe (sensitivity analysis; 1981-2021).

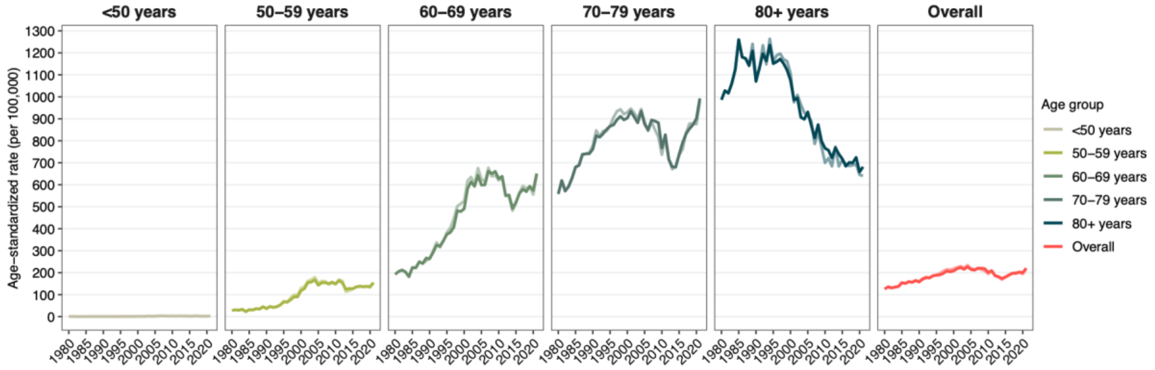

Legend: Rates per 100,000 men were directly age-standardized using the European 2013 standard population.

© 2026 Menges D et al. *JAMA Network Open*.

eFigure 6. Prostate Cancer Incidence by Prognostic Group After Excluding All Cases Receiving Prostate Surgery as Primary Treatment Due to Potential Upstaging (Sensitivity Analysis)

Data are presented by UICC stage (panels a-c) and Gleason score group (panels c-f) based on analyses using cases as registered and multiple imputation* (2002-2021). Upstaging through full pathology assessment of surgical specimen may have decreased over time as targeted biopsies were increasingly performed after approximately 2015.

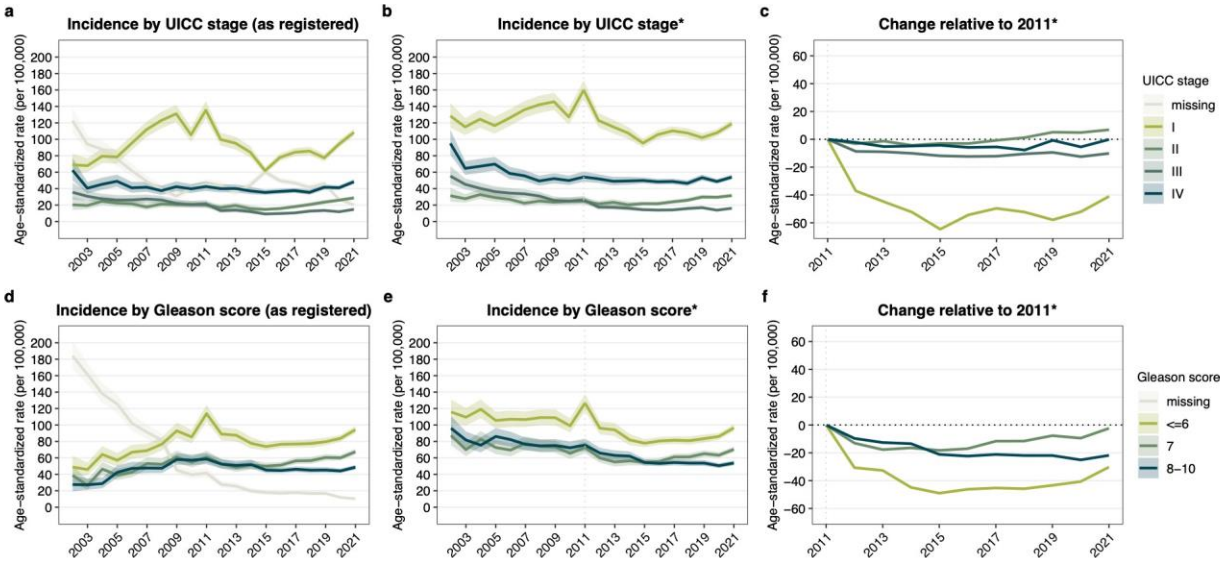

Legend: Rates per 100,000 men were directly age-standardized using the European 2013 standard population. Shaded areas represent 95% confidence intervals. * Multiple imputation was based on length of follow-up and status at end of follow-up of cases, the Nelson-Aalen estimator, incidence year, registry, and age group.

© 2026 Menges D et al. *JAMA Network Open*.

eFigure 7. Prostate Cancer Incidence by Prognostic Group After Excluding Cancer Registries Within Their First 2 Years of Registration or With ≥30% Missingness in a Given Year (Sensitivity Analysis)

Data are presented by SEER stage (panels a-c), UICC stage (panels d-f), and Gleason score group (panels g-i) based on cases as registered and based on multiple imputation* (2002-2021).

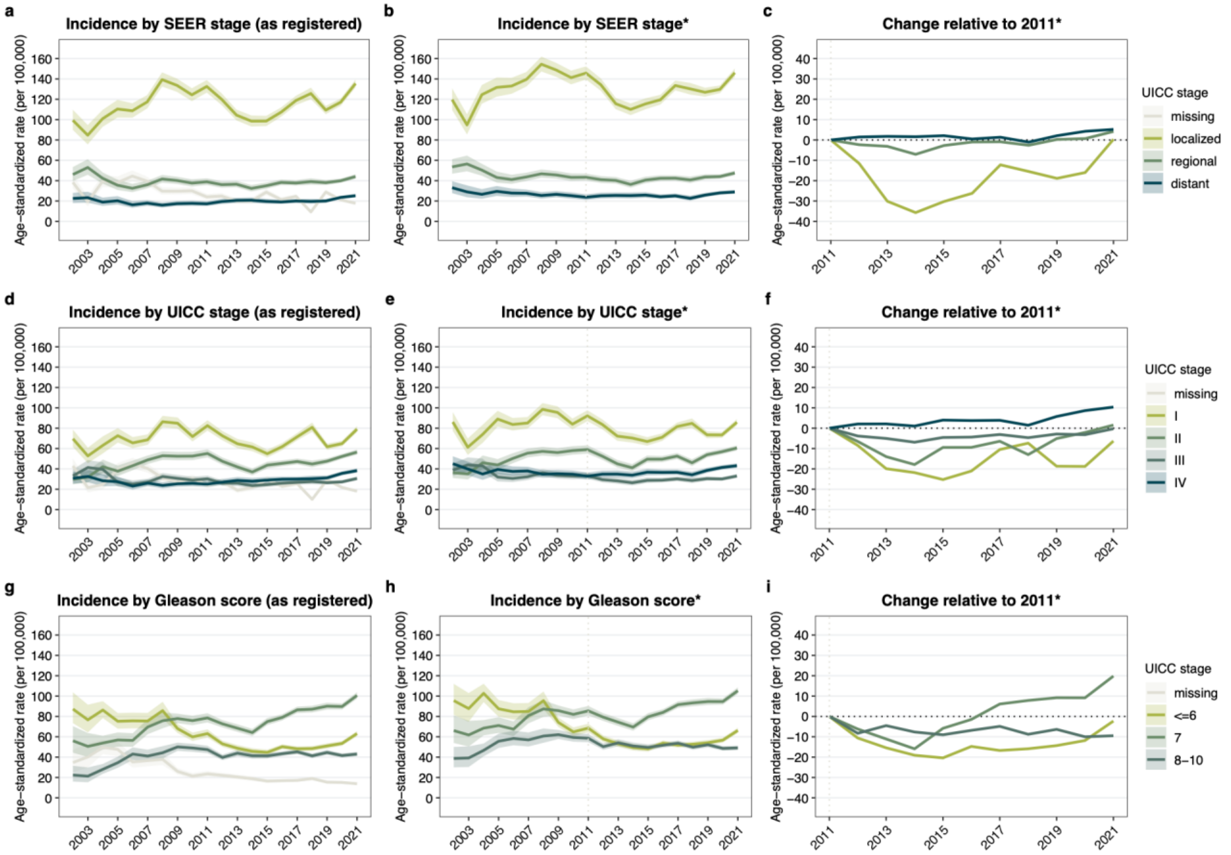

Legend: Rates per 100,000 men were directly age-standardized using the European 2013 standard population. Shaded areas represent 95% confidence intervals. * Multiple imputation was based on length of follow-up and status at end of follow-up of cases, the Nelson-Aalen estimator, incidence year, registry, and age group. Primary analyses presented in the article excluded all cancer registries within their first year of registration and all cancer registries with 100% missingness in respective risk variables.

© 2026 Menges D et al. *JAMA Network Open*.

eTable 7. Observed Prostate Cancer Deaths and Directly Age-Standardized Prostate Cancer Mortality Rates (ASMRs), Overall and by Age Group (1980-2021)

Incidence year	<50 years		50-59 years		60-69 years		70-79 years		80+ years		Overall	
	Obs. deaths	ASMR (95% CI)	Obs. deaths	ASMR (95% CI)	Obs. deaths	ASMR (95% CI)	Obs. deaths	ASMR (95% CI)	Obs. deaths	ASMR (95% CI)	Obs. deaths	ASMR (95% CI)
1980	0	0.0 (0.0 to 0.2)	32	9.5 (6.5 to 13.4)	146	56.4 (47.6 to 66.4)	395	234.6 (212.0 to 259.0)	368	721.2 (645.4 to 804.2)	941	64.9 (60.5 to 69.7)
1981	2	0.1 (0.0 to 0.3)	28	8.2 (5.5 to 11.9)	154	59.5 (50.5 to 69.6)	495	291.6 (266.4 to 318.6)	482	935.6 (849.3 to 1028.8)	1,161	81.0 (76.0 to 86.4)
1982	0	0.0 (0.0 to 0.2)	27	7.9 (5.2 to 11.5)	147	56.8 (48.0 to 66.8)	485	280.2 (255.7 to 306.4)	495	893.9 (812.3 to 982.1)	1,154	77.5 (72.7 to 82.6)
1983	0	0.0 (0.0 to 0.2)	21	6.1 (3.8 to 9.3)	168	65.5 (56.0 to 76.3)	464	265.1 (241.4 to 290.4)	470	831.8 (753.9 to 916.1)	1,123	73.8 (69.2 to 78.7)
1984	1	0.0 (0.0 to 0.2)	31	8.9 (6.1 to 12.7)	154	60.7 (51.5 to 71.2)	494	278.2 (254.2 to 303.9)	498	845.5 (768.6 to 928.5)	1,178	75.5 (70.9 to 80.4)
1985	0	0.0 (0.0 to 0.2)	27	7.7 (5.1 to 11.2)	168	65.4 (55.9 to 76.2)	553	306.9 (281.8 to 333.6)	577	967.6 (886.1 to 1055.0)	1,325	84.6 (79.7 to 89.7)
1986	0	0.0 (0.0 to 0.2)	31	8.8 (5.9 to 12.4)	190	71.8 (61.9 to 82.8)	481	266.3 (243.0 to 291.2)	561	894.8 (818.6 to 976.8)	1,263	78.1 (73.6 to 82.9)
1987	0	0.0 (0.0 to 0.2)	24	6.7 (4.3 to 10.0)	155	57.7 (48.9 to 67.5)	501	278.6 (254.7 to 304.1)	591	906.3 (831.3 to 986.6)	1,271	77.9 (73.4 to 82.6)
1988	0	0.0 (0.0 to 0.2)	18	5.0 (2.9 to 7.8)	188	68.5 (59.1 to 79.0)	521	290.2 (265.8 to 316.2)	632	920.7 (847.3 to 999.2)	1,359	80.7 (76.2 to 85.4)
1989	0	0.0 (0.0 to 0.2)	24	6.5 (4.2 to 9.7)	164	58.9 (50.2 to 68.6)	517	288.0 (263.7 to 314.0)	674	971.9 (897.1 to 1051.6)	1,379	82.2 (77.7 to 86.9)
1990	0	0.0 (0.0 to 0.2)	24	6.5 (4.1 to 9.6)	177	62.9 (54.0 to 72.9)	541	300.6 (275.8 to 327.1)	732	1001.8 (927.7 to 1080.4)	1,474	85.2 (80.8 to 89.9)
1991	0	0.0 (0.0 to 0.2)	34	9.0 (6.2 to 12.6)	157	55.5 (47.2 to 64.9)	511	282.9 (258.9 to 308.5)	732	954.7 (884.3 to 1029.5)	1,434	80.8 (76.5 to 85.3)
1992	0	0.0 (0.0 to 0.2)	31	8.2 (5.6 to 11.7)	174	61.7 (52.9 to 71.6)	538	297.3 (272.7 to 323.5)	706	919.0 (850.4 to 991.9)	1,449	80.9 (76.7 to 85.4)

© 2026 Menges D et al. *JAMA Network Open*.

Incidence year	<50 years		50-59 years		60-69 years		70-79 years		80+ years		Overall	
	Obs. deaths	ASMR (95% CI)	Obs. deaths	ASMR (95% CI)	Obs. deaths	ASMR (95% CI)	Obs. deaths	ASMR (95% CI)	Obs. deaths	ASMR (95% CI)	Obs. deaths	ASMR (95% CI)
1993	0	0.0 (0.0 to 0.2)	28	7.3 (4.8 to 10.5)	173	60.9 (52.1 to 70.7)	520	286.1 (262.0 to 311.9)	777	977.1 (907.7 to 1050.6)	1,498	82.6 (78.3 to 87.0)
1994	0	0.0 (0.0 to 0.2)	27	6.9 (4.5 to 10.0)	178	62.3 (53.5 to 72.2)	513	281.0 (257.1 to 306.5)	818	1005.8 (936.4 to 1079.3)	1,536	83.7 (79.4 to 88.1)
1995	0	0.0 (0.0 to 0.2)	37	9.2 (6.5 to 12.7)	153	52.7 (44.7 to 61.8)	435	233.1 (211.6 to 256.2)	675	807.8 (746.7 to 872.8)	1,300	68.7 (64.9 to 72.6)
1996	0	0.0 (0.0 to 0.2)	27	6.7 (4.4 to 9.7)	145	49.3 (41.6 to 58.0)	430	225.9 (205.0 to 248.4)	682	801.9 (741.7 to 865.8)	1,284	67.0 (63.3 to 70.9)
1997	0	0.0 (0.0 to 0.2)	29	6.9 (4.6 to 10.0)	146	49.6 (41.9 to 58.3)	451	231.3 (210.5 to 253.8)	645	752.6 (694.8 to 814.1)	1,271	65.1 (61.5 to 68.9)
1998	0	0.0 (0.0 to 0.2)	28	6.4 (4.3 to 9.3)	149	49.6 (42.0 to 58.3)	450	225.8 (205.4 to 247.7)	636	728.0 (671.8 to 787.7)	1,263	63.3 (59.8 to 67.0)
1999	0	0.0 (0.0 to 0.2)	30	6.9 (4.6 to 9.8)	156	51.1 (43.4 to 59.8)	491	240.7 (219.8 to 262.9)	682	772.3 (714.9 to 833.2)	1,359	67.1 (63.5 to 70.8)
2000	0	0.0 (0.0 to 0.2)	28	6.1 (4.0 to 8.8)	161	51.8 (44.1 to 60.5)	437	210.6 (191.3 to 231.4)	681	756.3 (700.1 to 816.1)	1,307	63.6 (60.1 to 67.2)
2001	0	0.0 (0.0 to 0.2)	31	6.6 (4.5 to 9.4)	148	47.3 (40.0 to 55.6)	453	215.3 (195.9 to 236.1)	685	729.7 (675.4 to 787.2)	1,317	62.2 (58.8 to 65.7)
2002	0	0.0 (0.0 to 0.2)	27	5.6 (3.7 to 8.2)	146	45.7 (38.6 to 53.8)	412	193.6 (175.3 to 213.2)	682	701.0 (648.7 to 756.5)	1,267	58.5 (55.3 to 61.9)
2003	0	0.0 (0.0 to 0.2)	30	6.2 (4.2 to 8.8)	140	42.9 (36.0 to 50.6)	414	191.9 (173.8 to 211.3)	732	736.5 (683.3 to 793.0)	1,316	59.9 (56.6 to 63.3)
2004	0	0.0 (0.0 to 0.2)	22	4.5 (2.8 to 6.8)	147	43.7 (36.9 to 51.4)	423	192.6 (174.7 to 211.9)	712	700.1 (648.7 to 754.6)	1,304	58.0 (54.8 to 61.3)
2005	0	0.0 (0.0 to 0.1)	30	6.1 (4.1 to 8.7)	112	33.4 (27.5 to 40.3)	402	179.4 (162.3 to 197.8)	718	675.9 (626.5 to 728.2)	1,262	54.6 (51.6 to 57.8)
2006	0	0.0 (0.0 to 0.1)	23	4.7 (3.0 to 7.0)	140	39.6 (33.3 to 46.8)	408	178.9 (161.9 to 197.1)	709	646.9 (599.5 to 697.1)	1,280	53.6 (50.7 to 56.7)

© 2026 Menges D et al. *JAMA Network Open*.

Incidence year	<50 years		50-59 years		60-69 years		70-79 years		80+ years		Overall	
	Obs. deaths	ASMR (95% CI)	Obs. deaths	ASMR (95% CI)	Obs. deaths	ASMR (95% CI)	Obs. deaths	ASMR (95% CI)	Obs. deaths	ASMR (95% CI)	Obs. deaths	ASMR (95% CI)
2007	0	0.0 (0.0 to 0.1)	26	5.3 (3.4 to 7.7)	137	37.5 (31.5 to 44.4)	382	164.3 (148.2 to 181.7)	716	633.3 (587.3 to 682.1)	1,261	51.5 (48.6 to 54.4)
2008	0	0.0 (0.0 to 0.1)	24	4.8 (3.1 to 7.2)	135	35.7 (29.9 to 42.3)	400	168.2 (152.1 to 185.5)	754	649.9 (603.9 to 698.6)	1,313	52.4 (49.6 to 55.4)
2009	1	0.0 (0.0 to 0.2)	21	4.2 (2.6 to 6.4)	126	32.0 (26.6 to 38.1)	380	156.4 (141.0 to 172.9)	746	615.6 (571.8 to 661.9)	1,274	49.1 (46.4 to 51.9)
2010	0	0.0 (0.0 to 0.1)	29	5.6 (3.8 to 8.1)	134	32.9 (27.5 to 39.0)	368	148.4 (133.6 to 164.4)	887	710.8 (664.4 to 759.6)	1,418	53.4 (50.7 to 56.3)
2011	0	0.0 (0.0 to 0.1)	19	3.6 (2.2 to 5.6)	152	36.4 (30.8 to 42.7)	366	144.7 (130.2 to 160.3)	827	652.2 (608.2 to 698.7)	1,364	50.3 (47.6 to 53.1)
2012	0	0.0 (0.0 to 0.1)	25	4.6 (3.0 to 6.8)	144	33.9 (28.6 to 39.9)	327	126.1 (112.8 to 140.6)	779	599.9 (558.1 to 644.0)	1,275	45.9 (43.4 to 48.5)
2013	0	0.0 (0.0 to 0.1)	29	5.2 (3.5 to 7.5)	143	33.2 (28.0 to 39.1)	348	130.4 (117.1 to 144.9)	832	620.9 (579.0 to 665.0)	1,352	47.3 (44.8 to 49.9)
2014	0	0.0 (0.0 to 0.1)	26	4.5 (2.9 to 6.6)	134	30.8 (25.8 to 36.5)	334	120.2 (107.7 to 133.9)	836	604.5 (563.9 to 647.3)	1,330	45.2 (42.8 to 47.7)
2015	0	0.0 (0.0 to 0.1)	22	3.7 (2.3 to 5.6)	126	28.7 (23.9 to 34.2)	378	131.8 (118.8 to 145.9)	829	579.4 (540.3 to 620.6)	1,355	44.6 (42.3 to 47.1)
2016	0	0.0 (0.0 to 0.1)	26	4.3 (2.8 to 6.3)	123	27.8 (23.1 to 33.2)	340	115.6 (103.6 to 128.6)	768	517.4 (481.2 to 555.6)	1,257	40.1 (37.9 to 42.4)
2017	0	0.0 (0.0 to 0.1)	26	4.1 (2.7 to 6.1)	125	28.3 (23.6 to 33.7)	367	119.4 (107.5 to 132.4)	903	592.1 (553.9 to 632.3)	1,421	44.2 (41.9 to 46.5)
2018	0	0.0 (0.0 to 0.1)	28	4.4 (2.9 to 6.4)	119	26.7 (22.1 to 32.0)	354	111.5 (100.2 to 123.8)	895	565.6 (529.0 to 604.1)	1,396	42.0 (39.8 to 44.3)
2019	0	0.0 (0.0 to 0.1)	23	3.5 (2.2 to 5.3)	128	28.5 (23.7 to 33.8)	386	117.2 (105.8 to 129.5)	839	512.4 (478.2 to 548.5)	1,376	39.9 (37.8 to 42.1)
2020	1	0.0 (0.0 to 0.2)	18	2.7 (1.6 to 4.3)	117	25.6 (21.2 to 30.8)	337	99.3 (89.0 to 110.5)	850	504.7 (471.3 to 540.0)	1,323	37.5 (35.5 to 39.6)

© 2026 Menges D et al. *JAMA Network Open*.

Incidence year	<50 years		50-59 years		60-69 years		70-79 years		80+ years		Overall	
	**Obs. deaths**	**ASMR (95% CI)**	**Obs. deaths**	**ASMR (95% CI)**	**Obs. deaths**	**ASMR (95% CI)**	**Obs. deaths**	**ASMR (95% CI)**	**Obs. deaths**	**ASMR (95% CI)**	**Obs. deaths**	**ASMR (95% CI)**
2021	0	0.0 (0.0 to 0.1)	22	3.3 (2.1 to 5.0)	106	23.0 (18.8 to 27.8)	351	101.0 (90.7 to 112.2)	780	452.4 (421.1 to 485.4)	1,259	34.8 (32.9 to 36.8)

Legend: ASMR = age-standardized mortality rate, CI = confidence interval, obs. deaths = observed deaths.

© 2026 Menges D et al. *JAMA Network Open*.

eTable 8. Relative Survival of Prostate Cancer Cases Across Incidence Periods, Overall and by Age Group (1980-2021)

Age group	Follow-up (years)	1980-1981 (%, 95% CI)	1982-1991 (%, 95% CI)	1992-2001 (%, 95% CI)	2002-2011 (%, 95% CI)	2012-2021 (%, 95% CI)
Overall	1	85.0 (81.8 to 88.3)	90.3 (89.2 to 91.5)	96.6 (96.2 to 97.0)	97.2 (96.9 to 97.5)	97.7 (97.5 to 97.9)
	5	50.9 (46.3 to 55.9)	62.8 (60.8 to 64.8)	83.5 (82.5 to 84.7)	90.0 (89.3 to 90.7)	92.2 (91.7 to 92.8)
	10	28.9 (24.9 to 33.5)	46.2 (43.7 to 48.8)	75.9 (73.8 to 78.0)	86.0 (84.6 to 87.4)	88.5 (86.6 to 90.5)
<50 years	1	83.0 (63.7 to 108.1)	87.4 (77.4 to 98.7)	99.4 (97.7 to 101.2)	100.0 (99.4 to 100.5)	99.3 (98.5 to 100.1)
	5	30.8 (12.9 to 73.4)	54.4 (40.4 to 73.2)	78.0 (70.4 to 86.4)	93.2 (90.3 to 96.2)	94.7 (92.4 to 97.0)
	10	32.0 (13.4 to 76.3)	38.5 (25.2 to 59.0)	68.5 (59.9 to 78.4)	90.4 (86.8 to 94.2)	91.7 (87.9 to 95.7)
50-59 years	1	89.9 (82.9 to 97.5)	94.4 (92.3 to 96.5)	98.6 (97.9 to 99.3)	99.4 (99.1 to 99.7)	99.7 (99.5 to 99.9)
	5	50.7 (39.6 to 64.8)	63.1 (58.9 to 67.5)	89.5 (87.8 to 91.1)	95.6 (94.8 to 96.4)	97.6 (97.1 to 98.2)
	10	28.1 (18.6 to 42.6)	46.9 (42.4 to 51.9)	81.2 (79.0 to 83.5)	92.3 (91.2 to 93.5)	95.6 (94.5 to 96.8)
60-69 years	1	91.7 (88.5 to 95.0)	94.4 (93.3 to 95.4)	98.5 (98.0 to 98.9)	99.7 (99.5 to 100.0)	99.8 (99.7 to 100.0)
	5	66.3 (60.5 to 72.5)	69.3 (67.3 to 71.5)	88.5 (87.5 to 89.6)	97.1 (96.6 to 97.7)	98.2 (97.8 to 98.7)
	10	42.5 (36.1 to 50.1)	54.0 (51.4 to 56.6)	81.7 (80.2 to 83.2)	95.2 (94.4 to 96.1)	96.0 (95.0 to 97.0)
70-79 years	1	86.7 (83.7 to 89.8)	92.5 (91.5 to 93.5)	97.2 (96.6 to 97.8)	99.0 (98.6 to 99.3)	99.4 (99.2 to 99.7)
	5	56.7 (51.7 to 62.3)	66.4 (64.5 to 68.4)	85.3 (83.9 to 86.6)	93.6 (92.6 to 94.5)	96.8 (96.1 to 97.5)
	10	33.1 (27.3 to 40.2)	49.6 (47.0 to 52.4)	76.0 (73.9 to 78.1)	92.0 (90.5 to 93.6)	96.2 (94.4 to 98.1)
80+ years	1	76.2 (70.1 to 82.8)	83.2 (81.3 to 85.2)	91.3 (90.0 to 92.7)	89.7 (88.5 to 90.9)	91.5 (90.6 to 92.3)
	5	39.7 (30.9 to 51.1)	57.5 (53.9 to 61.4)	72.2 (69.2 to 75.2)	70.6 (68.1 to 73.1)	74.3 (72.3 to 76.4)
	10	19.6 (9.4 to 41.0)	42.0 (35.7 to 49.3)	62.5 (57.0 to 68.7)	58.8 (54.4 to 63.6)	62.2 (56.2 to 68.9)

Legend: CI = confidence interval.

© 2026 Menges D et al. *JAMA Network Open*.

eFigure 8. Relative Survival of Prostate Cancer Cases by Prognostic Group and Age Group (2002-2021)

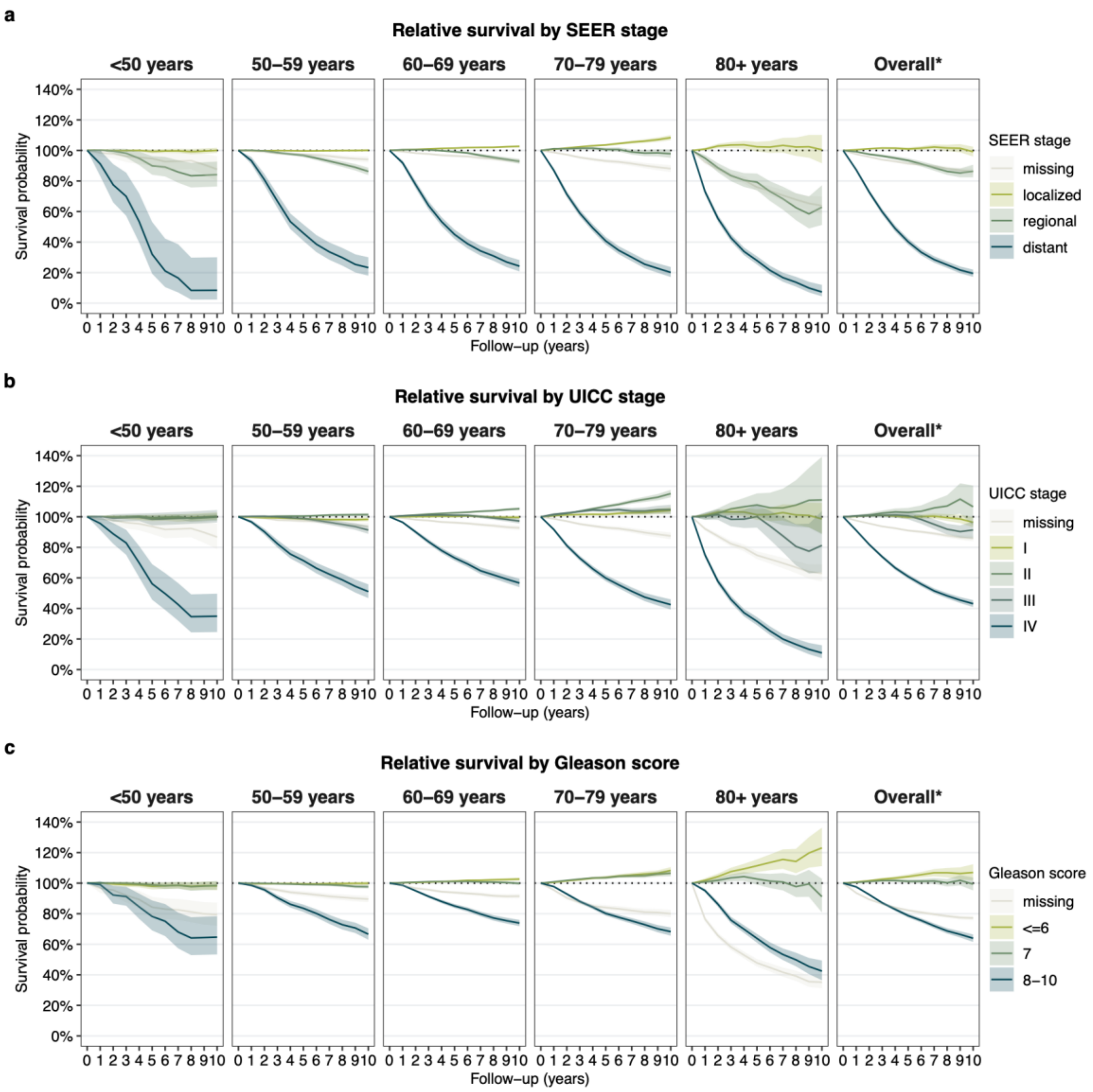

Legend: Relative survival was estimated relative to the expected mortality among men from the Swiss general population. Shaded areas represent 95% confidence intervals. Cases with missing information were treated as separate category in subgroup analyses. Relative survival proportions greater than 100% represent better survival compared to general population. * Overall relative survival proportions were age-standardized using the ICSS1 standard population.

© 2026 Menges D et al. *JAMA Network Open*.

eFigure 9. Relative Survival of Prostate Cancer Cases by SEER Stage and Incidence Period (2002-2021)

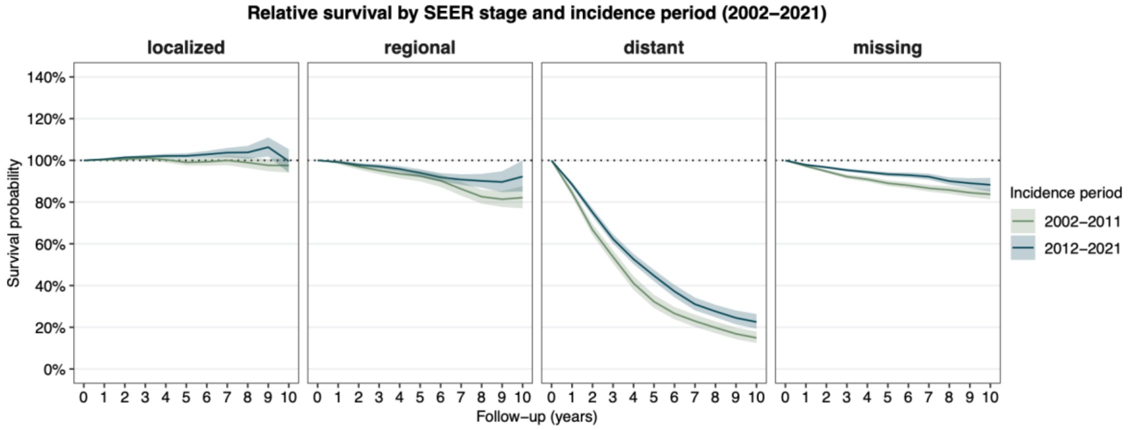

Legend: Relative survival was estimated relative to the expected mortality among men from the Swiss general population. Shaded areas represent 95% confidence intervals. Cases with missing information were treated as separate category. Relative survival proportions greater than 100% represent better survival compared to general population. Relative survival proportions were age-standardized using the ICSS1 standard population.

© 2026 Menges D et al. *JAMA Network Open*.

eFigure 10. External Evidence on Prostate Cancer Testing From Swiss Data Sources

Data are presented for timing of last prostate cancer examination[a] according to the Swiss Health Survey (2012-2022; panel a) and prostate-specific antigen (PSA) testing according to the Swiss Healthcare Atlas (2013-2021; panel b) in Switzerland.

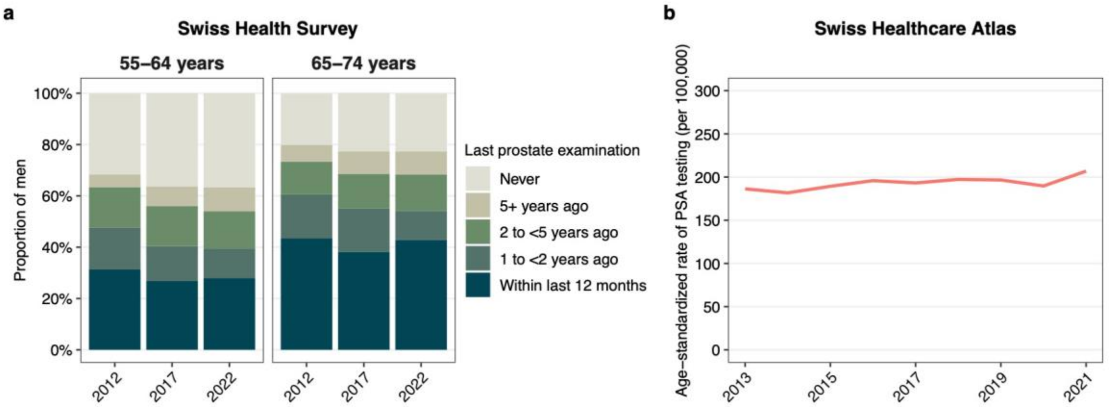

Legend: PSA = Prostate-specific antigen. [a] Prostate examination was defined as either a PSA test or a digital rectal examination for any reason. Data related to prostate cancer examinations from the Swiss Health Survey are publicly available and were obtained from https://www.bfs.admin.ch/asset/de/30305992 (accessed 17 Jan 2025).
Data related to PSA testing from the Swiss Healthcare Atlas are publicly available and was obtained from https://www.versorgungsatlas.ch/en/indicator/_088/a (accessed 15 Jan 2025).
Data visualizations were produced by the authors.

© 2026 Menges D et al. *JAMA Network Open*.

eReferences

1. Surveillance, Epidemiology, and End Results Program (SEER). SEER Summary Stage 2018 - Male Genital System (Version 3.2). 2024. https://seer.cancer.gov/tools/ssm/SSM2018-MALE-GENITAL-SYSTEM.pdf. Accessed January 13, 2025.
2. Brierley JD, Gospodarowicz MK, Wittekind C. TNM Classification of Malignant Tumours, 8th Edition. Wiley-Blackwell; 2017.
3. Epstein JI, Allsbrook WCJ, Amin MB, Egevad LL, Committee and the IG. The 2005 International Society of Urological Pathology (ISUP) Consensus Conference on Gleason Grading of Prostatic Carcinoma. The American Journal of Surgical Pathology. 2005;29(9):1228. doi:10.1097/01.pas.0000173646.99337.b1
4. Rubin DB. Multiple Imputation for Nonresponse in Surveys. Vol 81. John Wiley & Sons; 2004.
5. Ng HKT, Filardo G, Zheng G. Confidence interval estimating procedures for standardized incidence rates. Computational Statistics & Data Analysis. 2008;52(7):3501-3516. doi:10.1016/j.csda.2007.11.004

© 2026 Menges D et al. *JAMA Network Open*.
