# Supplementary material for: Prostate Cancer Incidence, Mortality, and Survival in Switzerland
Source: JAMA Netw Open. 2026 Apr 21;9(4):e268289. doi: 10.1001/jamanetworkopen.2026.8289 (PMC13100841; doi:10.1001/jamanetworkopen.2026.8289)
Supplement: Supplement 2. — Data Sharing Statement [file jamanetwopen-e268289-s002.pdf]

Data Sharing Statement

Menges. Prostate Cancer Incidence, Mortality, and Survival in Switzerland. *JAMA Netw Open*. Published April 21, 2026. doi:10.1001/jamanetworkopen.2026.8289

Data
Data available: No

Additional Information
Explanation for why data not available: All data used in this work are held by the Swiss National Agency for Cancer Registration (NACR) and the Swiss Federal Statistical Office (SFSO). The contracts with these entities preclude the direct sharing of the original data by the authors. Data can be obtained through direct request to the NACR for cancer registration data (https://nacr.ch/en/information-for-researchers) and the SFSO for mortality data (https://www.bfs.admin.ch/bfs/en/home/statistics/health/surveys/ecod.html), respectively.
